# From mechanism to application: Decrypting light‐regulated denitrifying microbiome through geometric deep learning

**DOI:** 10.1002/imt2.162

**Published:** 2024-01-06

**Authors:** Yang Liao, Jing Zhao, Jiyong Bian, Ziwei Zhang, Siqi Xu, Yijian Qin, Shiyu Miao, Rui Li, Ruiping Liu, Meng Zhang, Wenwu Zhu, Huijuan Liu, Jiuhui Qu

**Affiliations:** ^1^ Center for Water and Ecology, State Key Joint Laboratory of Environment Simulation and Pollution Control, School of Environment Tsinghua University Beijing China; ^2^ Department of Computer Science and Technology Tsinghua University Beijing China; ^3^ School of Electronic and Information Engineering Beihang University Beijing China

**Keywords:** denitrification, graph neural networks, meta‐omics, microbiomes, optogenetics

## Abstract

Regulation on denitrifying microbiomes is crucial for sustainable industrial biotechnology and ecological nitrogen cycling. The holistic genetic profiles of microbiomes can be provided by meta‐omics. However, precise decryption and further applications of highly complex microbiomes and corresponding meta‐omics data sets remain great challenges. Here, we combined optogenetics and geometric deep learning to form a discover–model–learn–advance (DMLA) cycle for denitrification microbiome encryption and regulation. Graph neural networks (GNNs) exhibited superior performance in integrating biological knowledge and identifying coexpression gene panels, which could be utilized to predict unknown phenotypes, elucidate molecular biology mechanisms, and advance biotechnologies. Through the DMLA cycle, we discovered the wavelength‐divergent secretion system and nitrate‐superoxide coregulation, realizing increasing extracellular protein production by 83.8% and facilitating nitrate removal with 99.9% enhancement. Our study showcased the potential of GNNs‐empowered optogenetic approaches for regulating denitrification and accelerating the mechanistic discovery of microbiomes for in‐depth research and versatile applications.

## Highlights

- Graph neural networks (GNNs)‐based biology‐contextualized computational framework exhibited superior performance in identifying coexpressed gene panels and decrypting wavelength‐dependent denitrification.
- Wet‐lab demonstrations validated the wavelength‐divergent secretion system and nitrate‐superoxide co‐regulation as unveiled by GNNs, which could be utilized for nitrate removal and resource recovery.
- The coexpressed gene panels and topological network toolkits were developed to guide scientific discovery and versatile biotechnology development.

## Graphical abstract

This study proposed and showcased the discover–model–learn–advance (DMLA) cycle for unlocking nature as codebases to empower biological mechanisms decryption and biotechnology development. At the discovering stage, we found the bidirectionally light‐regulated denitrification. Then, metatranscriptomic was utilized for geometric deep learning to obtain coexpressed gene panels. On the basis of that, we developed toolkits to learn underlying mechanisms and advance biotechnology, which were validated in the wet lab. Deepening recognition can further drive continuous DMLA cycles to accelerate scientific and biotechnology development.

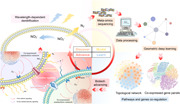

## INTRODUCTION

Denitrifying microbiome is essential in maintaining nitrogen cycling in the ecosystem, mostly through denitrification that reduces nitrate and nitrite to gaseous forms of nitrogen, as well as converts nitrate to ammonia and organic nitrogen [1]. The planetary nitrogen boundary has raised extensive concerns and is estimated to have reached a high‐risk zone [2]. Moreover, denitrifying microbiome also played a pivotal role in socioeconomic development, such as food production [3], energy [4, 5], wastewater treatment [6], and resource recovery [7]. For example, denitrification is widely used in nitrate removal for the toxicity of nitrate on both human and aquatic organisms, which takes a considerable proportion of costs in industrial production. Therefore, various approaches have been proposed to regulate denitrification, including adding conductive materials [8], applying magnetic field and light illumination [9, 10], appropriate bioreactor designing and operations [11], and so forth. Among these strategies, optical technology is promising for its superior advantages, including low costs, environment‐friendly, solar‐to‐chemical capability, selectivity, and precise microbial control [12, 13, 14]. This is because light can serve as signals of optogenetic switches to modulate cellular activities, like, light‐sensitive promoters, ion channels, pumps, and protein conformation variations [15]. Recently, we succeeded in employing light wavelengths to bidirectionally regulate denitrifying sludge for different economic nitrate removal processes [10].

For all the regulatory strategies, meta‐omics has emerged as a valuable approach to provide genetic information of microbiomes, including high‐dimension data on species, genes, proteins, metabolism pathways, species, and so forth [16]. However, decrypting biological big data requires sophisticated skills and highly professional biological knowledge. In addition, searching for targeted functions is laborious and the selection of targeted functions is subjective, leading to the proposed mechanism scheme hard to demonstrate in the wet lab and scale‐up. Furthermore, the conventional approaches usually focus on individual gene or enzyme, hard to capture the dynamic biological networks that are systematically correlated on multilevels [17], such as genetic, metabolic, and cellular levels. It is especially challenging for environmental microbiota given its complexity and cross‐species interactions.

Deep learning exhibits superior performance in characterizing biological big data and learning genetic topological and coexpression principles [18]. Among them, multilayer perception, convolutional neural network, and long short‐term memory are mostly used [19]. However, these methods cannot elaborate the multilevel topological information of biological systems and are also limited to Euclidean data sets [20]. Recently, geometric deep learning has received great attention for its great representing capability in non‐Euclidean data sets [21], among which graph neural network (GNN) is the most representative and successful one in exploiting heterogeneous information and complex topological relationship [22, 23].

**Figure 1:**
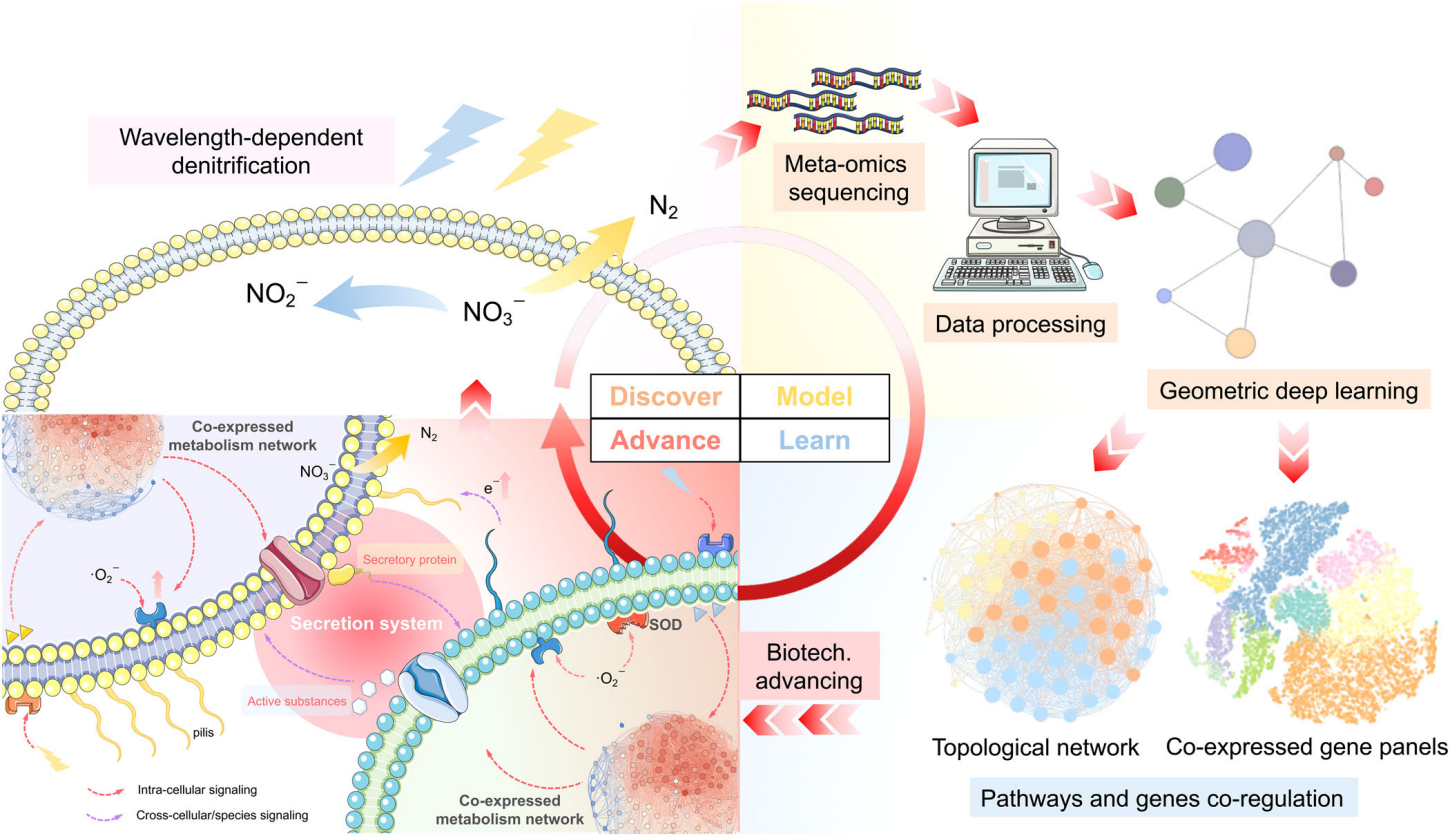
Schematic illustration on the discover–model–learn–advance (DMLA) cycle for microbiome technology development. In this study, we discovered that light wavelengths can be utilized to bidirectionally regulate bio‐denitrification to nitrogen gas or nitrite for different nitrate removal strategies. After that, we conducted metatranscriptomic sequencing and data preprocessing to obtain graph‐structured data sets for modeling. Graph neural networks, a representative geometric deep‐learning approach, were utilized to unsupervisedly learn the gene panels. On the basis of the critical gene panels, we learned the coexpressed pathways and genes through the model toolkits we proposed and validated the knowledge we learned in the wet lab, which drove the biotechnology advancement, including new applications and mechanism discovery. The new mechanism deepened our recognitions on optogenetics in microbiomes that wavelength‐dependent secretion systems played a pivotal role in the collective behavior of microbiota in response to light wavelength. The secreted active substances and proteins mediated the cross‐cellular interactions.

Here, we showcased adopting geometric deep learning to decrypt meta‐omics data sets of environmental microbiota. After discovering the light‐regulated denitrification in the lab, that is, the discovery stage in Figure 1, we followed the discover–model–learn–advance (DMLA) cycle to deepen our understanding of the optogenetic mechanism and advance its applications. At the modeling stage, we exploited GNNs and Deep Graph Infomax (DGI), an unsupervised deep‐learning algorithm for graph‐structured data sets [24], to integrate gene expression and subcellular information for gene panel identification. Guided by the developed toolkits at the learning stage, we extend the applications of optical biotechnology, including increasing extracellular protein production (83.3% higher) and recovering light‐induced bioactive substances. Also, the signaling role of superoxide achieved nitrate removal enhancement by 99.9%. The mechanism scheme was also reconstructed at the advancing stage. As suggested by the divergent self‐catalytic effects, the secretion system played an essential role in wavelength‐dependent nitrogen metabolism. Pathway enrichment and topological analysis revealed that yellow light centralizes metabolism fluxes to synthesize more proteins, such as pilis and molecular chaperones. Contrariwise, blue light decentralized the metabolism fluxes to secrete rare bioactive substances. Photons with different frequencies, light‐induced second messengers, and superoxide may serve as a signal to promote substance exchanges and collective cellular metabolism. Besides denitrifying microbiomes, we also extended the applications to other acclimated microbiomes and practical engineering biosamples. Our work offered a framework for microbiome decryption and demonstrated the potential of GNN‐empowered optical biotechnology in regulating microbial nitrogen metabolism.

## RESULTS

### Discovery of light‐regulated microbial metabolism and modeling meta‐omics through GNNs

**Figure 2:**
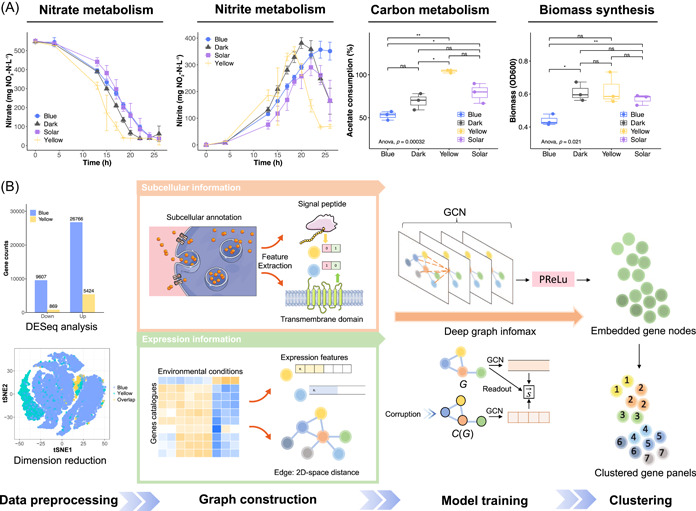
Overview of the geometric deep‐learning workflow for modeling wet‐lab discoveries. (A) Wet‐lab discoveries on light‐regulated nitrogen metabolism, carbon metabolism, and biomass synthesis of the microbiome. *p < 0.05; **p < 0.01; ns, not significant. Red arrows highlighted the comparisons mentioned. The significance analysis on acetate consumption and biomass was conducted at the end of photo‐denitrification (24 h). (B) The workflow of identifying coexpressed gene panels through GNNs. In data preprocessing, we first obtained differentially expressed genes (DEGs) through DESeq analysis. Then, we filtered low‐expression genes to obtain valid DEGs. The overlap in dimension reduction denoted DEGs shared by blue and yellow light data sets. In graph construction, we characterize subcellular information with 1 and 0 to represent whether or not individual genes encode signal peptides and possess transmembrane domains. Log normalization was performed on the expression level of valid DEGs to represent expression information. In model training, we adopted the graph convolutional network (GCN) as the GNN architecture, and utilized Deep Graph Infomax (DGI) algorithm for unsupervised learning to obtain node embedding. $\vec{S}$ is the summary vector. After that, the embedded gene nodes were clustered to obtain coexpressed gene panels which were utilized for mechanism elucidation, phenotype prediction, and biotechnology development. DESeq, differential expression; GNNs, graph neural networks; PReLu, parametric rectified linear unit; tSNE, T‐distributed stochastic neighbor embedding.

Solar light inhibition is prevalent for environmental microbiota, which hinders nitrate removal of wastewater [25]. While in the wet‐lab experiments, we found that the effects of single‐wavelength lights on activated sludge varied (Figure 2A). We adopted blue Light Emitting Diodes (LEDs) peaked at 456.2 nm and yellow LEDs peaked at 589.4 nm (Figure S1) to regulate the aquatic denitrifying microbiome. Overall, blue and yellow lights decomposed the inhibitory effects of solar light. Blue light exhibited inhibitory effects on microbial metabolism, realizing partial denitrification (PD) with a 69.4% nitrite accumulation ratio (NAR) at 26 h, much higher than dark (33.6% NAR). The stable nitrite accumulation was favorable for PD‐coupled anaerobic ammonium oxidation (PD/A), a much more economic nitrogen‐containing wastewater treatment compared with traditional denitrification [25]. Contrarily, yellow light boosted both nitrate and nitrite removal. Interestingly, yellow light also promoted carbon source intake by 49.5% compared with the dark, but no significant increase in biomass synthesis, indicating microbiota utilized acetate for other metabolism. In comparison, blue light reduced biomass accumulation by 27.8% compared with the dark, but no significant reduction in carbon source intake, which also implied the metabolism fluxes diversion from biomass synthesis. Bacterial viability staining revealed activated metabolism in both blue and yellow light groups, as opposed to dark and ultraviolet (UV) groups (Figure S2), further supporting the metabolism redirection.

To uncover the microbial transcriptional responses to light wavelengths, we conducted metatranscriptomics after photo‐denitrification. There were 56,991 nonredundant genes across all samples. First, we conducted data preprocessing and obtained 25,886 valid differentially expressed genes (DEGs). Data sets exploratory analysis, including differential expression (DESeq) analysis, dimension reduction, and hierarchical clustering (HC) of gene expression patterns (Figure S3), revealed that blue light triggered more substantial transcriptional divergence than yellow light and decentralized metabolic fluxes (Text S1). This was because a large number of genetic regulatory activities are responsive to blue light, such as genes that encode photoreceptor, promoter, and enhancer [12, 13]. While yellow light exhibited higher selectivity for a smaller gene set due to few genes reported to be responsive to yellow light [12, 13]. Moreover, the DEGs overlap in dimension reduction through T‐distributed stochastic neighbor embedding (tSNE) demonstrated the genetic coexpression.

Given the prior‐knowledge‐based database classification failed to obtain coexpressed gene panels (Figure S4), we adopted geometric deep learning on graph‐structured data sets to build contextually customed models (Figure 2B). Compared with the widely used single‐cell data sets, meta‐omics data sets were characterized by microbial interactions and frequent extra‐ and intracellular substance changes, thus hard to decrypt simply through linear regression or traditional machine learning given the environmental data noise. Thus, we integrated biological knowledge through graph convolutional networks (GCNs) to assist models in unsupervised learning regulatory networks of environmental microbiota, unleashing the enormous potential of the nature code base. After data processing, we obtained valid DEGs, whose subcellular information and expression information were engineered into graph‐structured data sets as described in Methods. Expression information characterized the intracellular regulatory networks, whereas subcellular information, including signal peptides and transmembrane domains, represented the intercellular interaction. We employed the DGI algorithm to unsupervisedly learn the node embeddings, which were then clustered to obtain the gene panels.

### Geometric deep‐learning achieves superior performance in gene panel identification

The determination of the number of coexpressed gene panels needs to rely on contextual biological knowledge. In our case, light‐wavelength bidirectionally regulated nitrate conversion, implying that genes related to light signaling, that is, phototransduction [26], would be coexpressed. This was further confirmed by similar gene expression patterns in response to illumination conditions (Figure S6A). In comparison, hub metabolism pathways, such as nitrogen metabolism, involved multifunctions [1, 27], presenting divergent expression patterns and thus not coexpressed (Figure S6B). Hence, we compared the cluster assignment of phototransduction genes (Figure S7) and defined seven coexpressed gene panels (Text S2).

**Figure 3:**
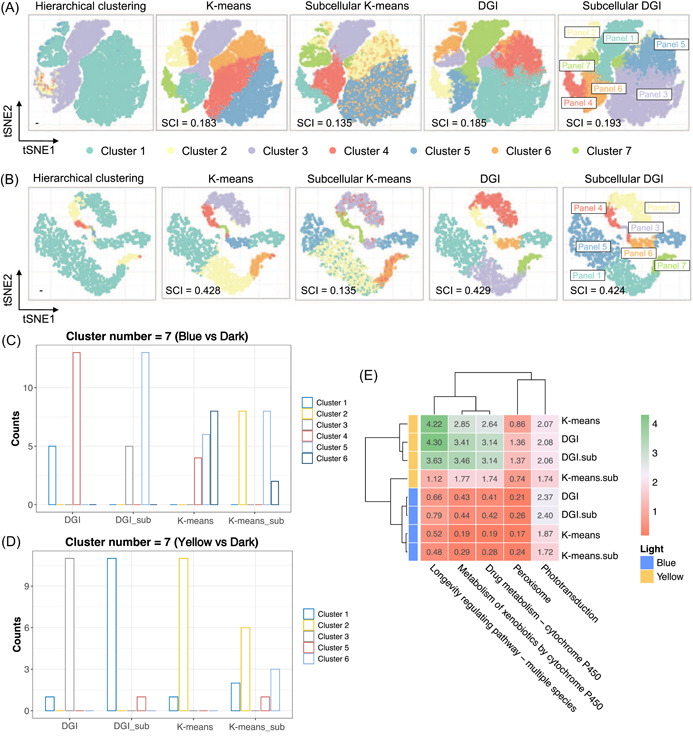
Evaluation on model clustering capability. (A, B) Two‐dimensional (2D) projection and clustering capability evaluation of unsupervised learned clusters of blue light (A) and yellow light (B) data sets. Classification capability was evaluated both qualitatively by cluster visualization in 2D T‐distributed stochastic neighbor embedding (tSNE) space and quantitively by Silhouette Coefficient Index (SCI), an intercluster similarity indicator. “Subcellular” indicated that the subcellular information was integrated. (C, D) Cluster assignments of genes related to phototransduction induced by of blue (C) and yellow light (D) with cluster number as 7. Counts indicate gene counts assigned to the cluster. “_sub”, integrated with subcellular information. (E) Functional assignment score (FAS) of light‐responsive pathways of different clustering approaches based on prior knowledge.

Genes in the same gene panel, that is, cluster, were genetically coregulated in response to blue and yellow light. Thus, to mathematically evaluate the gene coregulation identification capability in genetic latent space, we projected the clustering results of node embeddings on two‐dimensional (2D) latent space, as well as the dimension reduction results of other commonly used approaches without geometric deep learning (Figure 3A,B). HC presented strong biases that tended to cluster most genes into two clusters, suggesting poor performance in biological coexpression capturing. Compared with HC, both *K*‐means and DGI perform well in clustering coexpressed genes. However, *K*‐means failed to integrate heterogeneous information as suggested by the 2D distributions. It was obvious that unsupervised pretraining through DGI learned clear gene panels based on gene expression and subcellular information (Subcellular DGI in Figure 3A,B). Contrarily, some genes failed to cluster together and scatter around the latent space when only applying *K*‐means for heterogeneous information (Subcellular *K*‐means in Figure 3A,B), indicating it failed to capture optogenetic gene coregulation. Silhouette Coefficient Index (SCI), an intercluster similarity indicator, further confirmed the results with higher scores (Text S2).

As for the cluster assigning accuracy with regard to biological meaning, the DGI model outperformed *K*‐means regardless of cluster number (Figures S7 and 3C,D). DGI tended to cluster phototransduction genes into 1–2 clusters. In contrast, *K*‐means scattered genes across multiple clusters. These implied that DGI succeeded in integrating subcellular information and capturing genetic coexpression. To quantitively evaluate the biological functions matching performance, we defined functional assignment score (FAS). On the basis of prior knowledge [10], we compared the FAS of pathways that are closely related to light, including oxidative stress and optogenetic switches (Figure 3E). Generally, DGI with subcellular information possessed higher FAS, demonstrating that DGI outperformed *K*‐means and the integration of subcellular information assisted to identify biological functions (Text S2).

### Predicting phenotypes through differential pathways in the hub gene panel (HGP) and signaling gene panel (SGP)

**Figure 4:**
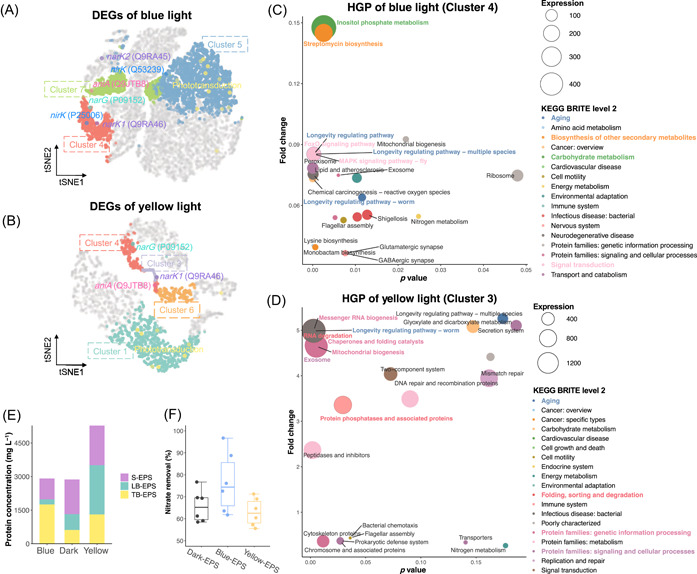
Decrypting hub gene panels (HGPs) and signaling gene panels (SGPs) to predict phenotype and the corresponding wet‐lab validations. (A, B) Spatial distribution of functional clusters and significant photo‐denitrification genes of blue (A) and yellow light (B). (C, D) Pathways enrichment analysis of HGPs of blue (C) and yellow (D) light. The most highly expressed pathways and their KEGG BRITE (KEGG database) were highlighted by corresponding color and bold font. Bubble size denoted mean expression levels (FPKM) under blue or yellow light, respectively. Fold changes were calculated with the dark group as the control. (E) Protein concentrations of stratified extracellular polymer substances (EPSs) under different illumination conditions. The thin white lines on the stacked bar separated the results of triplicates. (F) Self‐catalysis experiments. EPS under dark, blue, and yellow light conditions were added to the model denitrifier systems. DEG, differentially expressed gene; KEGG, Kyoto Encyclopedia of Genes and Genomes; LB‐EPS, loosely bound EPS; S‐EPS, soluble EPS; TB‐EPS, tightly bound EPS; tSNE, T‐distributed stochastic neighbor embedding.

The clusters obtained through DGI were coexpressed gene panels (i.e., clusters), which could be used to decrypt genetic mechanisms [28]. We developed gene panel toolkits (data and code availability) to unlock the natural code base for mechanism decryption and biotechnology development. After searching the annotations, it can be observed most of phototransduction genes were assigned to the same gene panel, cluster 5 for blue light and cluster 1 for yellow light (Figure 4A,B). In contrast, other clustering approaches (Figure 2A,B) would misallocate those genes to different clusters, which further confirmed the effectiveness of our model. As for nitrate‐ and nitrite‐related genes, that is, PD genes, most of them were mainly assigned to clusters 7 and 4 for blue light, and clusters 6, 3, and 4 for yellow light. We compared these clusters through enrichment analysis (Figure S9). We employed Fragments Per Kilobase of exon model per Million mapped fragments (FPKM) values to quantify the gene expression levels. For blue light, genes of cluster 4 contained more denitrification genes and were characterized with high expression levels and low fold changes, likewise cluster 3 for yellow light. Those clusters are regarded as HGPs [29]. On the contrary, clusters phototransduction genes subjected to were divergent from HGPs, exhibiting relatively high fold change and low expression. Given the signaling role of phototransduction, we defined those clusters as SGPs.

HGPs correspond to the collective behavior of microbiota, that is, phenotype, which could potentially be characterized in labs and harnessed for developing new biotechnology. Among the highly expressed HGPs' pathways, Aging, a level 2 KEGG BRITE (KEGG database) that contributes to cellular fitness and longevity in response to genetic and environmental stimulation (Text S3), was shared by both blue and yellow light (Figure 4C,D). Longevity‐regulating pathways were the predominant Aging pathways, which were characterized by highly active oxidative activities that would produce a large number of reactive oxygen species (ROS) [30], indicating that photo‐denitrification was coexpressed with ROS metabolism. We conducted wet‐lab validations on phenotypes of light‐induced ROS production (Figure S10). The total ROS levels of all groups increased in the nitrate reduction stage and decreased in the latter stages, taking on similar trends with nitrite concentrations. Additionally, both blue and yellow light promoted total ROS production, which was attributed to the photochemical stimulation of microbiota [31].

Signaling transduction was another evident Brite in HGPs of blue light, including FoxO signaling and MAPK signaling pathways, implying the predominant role of signaling under blue light irradiation. Carbohydrate metabolism and biosynthesis of other secondary metabolites dominated metabolism fluxes, especially inositol phosphate metabolism (490.85 FPKM), almost twice that of the second one. Inositol phosphate metabolism is an important hub that coordinates the growth factor signaling, energy homeostasis with nutrient uptake and utilization [32], implying that the higher levels of signaling substances produced by the microbiome regulated the nitrate uptake and conversion, potentially for survival and competition (Text S3). Contrariwise, yellow light's HGP was dominated by pathways for diverse protein synthesis (Figure 4D), including proteins related to genetic information processing, metabolism, signaling, cellular process, and so forth.

The overall expression levels of HGPs and SGPs comparison between blue and yellow light revealed metabolism flux redirections. Yellow light's HGP was significantly upregulated and much higher than blue light's, suggesting that the metabolism fluxes under yellow light were mostly redirected to HGPs to synthesize the proteins mentioned above. In contrast, the average expression level of blue light's SGP was much higher (15.70 FPKM) than yellow light's (3.66 FPKM) (Table S4), which explained the sluggish denitrification and acetate uptake, behaviors related to HGPs under blue light in Figure 1. The metabolism fluxes under blue light were redirected to SGPs for vital metabolites synthesis, such as secondary metabolites and glycan biosynthesis, metabolism of cofactors, and vitamins (Figure S11). These metabolites were valuable bioproducts with diverse biological functions, such as energy metabolism, intercellular signaling, cellular resistance, and protection, to maintain basic cellular function to survive under environmental stimulation (Text S4). Notably, the pathway of the secretion system was presented in blue light's SGP with significant expression (Figure S11). For yellow light instead, the secretion system was assigned to the HGP of yellow light (Figure 4D). This suggested the divergent role of secretion systems under blue light and yellow light irradiation. All in all, blue light triggered the secretion of bioactive substances, such as secondary metabolites, cofactors, and vitamins. Whereas yellow light contributed to the increased synthesis and secretion of proteins.

To validate the divergent secretion system, we extracted the extracellular polymer substances (EPSs) of microbiota after photo‐denitrification. As anticipated, the total protein concentration of yellow light ranked the highest and was increased by 83.8% compared with the dark control (Figure 4E), corresponding to the highly expressed pathways related to protein synthesis in Figure 4D. It suggested that yellow light potentially could be harnessed to induce protein production with nitrate wastewater as substrate. Additionally, blue light increased the proportion of tightly bounded EPS, corresponding to the significantly upregulated biosynthesis of exopolysaccharide and galactose metabolism (Figure S11A), which could facilitate the formation of biofilm to protect cells through exopolysaccharide, a kind of extracellular carbohydrate polymers [33, 34]. Furthermore, we utilized the extracted EPS as biocatalysts to demonstrate the divergent secreted substances under blue and yellow light. It turned out that the extracted EPS can promote nitrate removal with an average enhancement of 16.6% compared with dark (Figure 4F), which was consistent with the coexpression model above that microbiota under blue light secreted more bioactive substances to survive under photochemical oxidative stress [33].

### Regulating microbiome through landmark genes of topological networks

After discovering the unknown potential of microbiomes, such as using nitrate as substrates for bioproduction, precise regulatory strategies are necessary to enhance the bioreaction. Traditional approaches usually target individual genes or pathways, named biomarkers, which perform poorly due to a lack of system biology principles. Thus, we coupled the network topology [17] and landmark genes [28] to construct topological networks for precise gene regulation.

**Figure 5:**
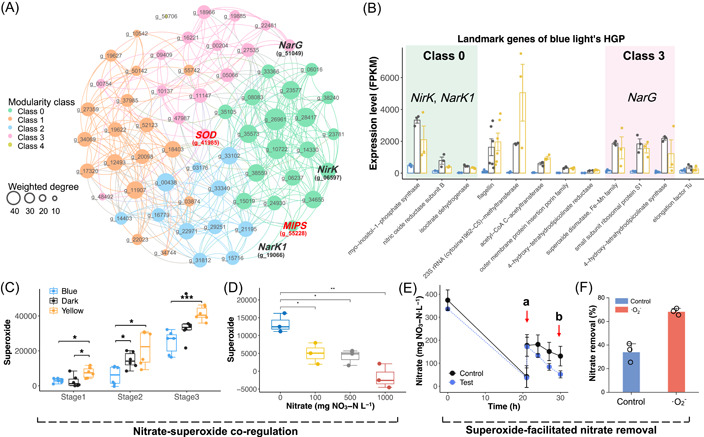
Exploiting landmark genes of gene topological networks to develop a regulatory strategy on nitrate reduction. (A) The gene topological network of blue light's HGP. Details on the topological information of gene nodes and landmark genes were summarized in Dataset S1 to S6. The bold red font highlighted the most highly expressed landmark genes. The bold black font highlighted the PD genes. (B) Expression levels of landmark genes of blue light's HGP. Background highlights the modularity class that crucial denitrification genes are subjected to. The different background colors represented different modularity classes. (C) Stage 1, nitrate reduction; Stage 2, nitrite reduction; Stage 3, Inorganic nitrogen depletion. (D) Superoxide production under different initial nitrate concentrations. (E) Superoxide supplementation experiment. At timepoint a, nitrate and superoxide were added. Timepoint b was used to calculate the effects of superoxide on nitrate removal efficiency. Both control and superoxide groups were conducted under dark conditions. (F) Effect of superoxide supplementation on nitrate removal efficiency. *DapA*, 4‐hydroxy‐tetrahydrodipicolinate synthase; HGP, hub gene panel; *IDH*, isocitrate dehydrogenase; *MIPS*, myo‐inositol‐1‐phosphate synthase; *NarG*, nitrate reductase; *NarK1*, nitrate/nitrite transporter; *NirK*, nitrite reductase; *NorB*, nitric oxide reductase subunit B; PD, partial denitrificatio; *SOD*, superoxide dismutase.

To obtain high‐credit gene representations, we conducted modularity classification within gene panels to obtain subnetwork and corresponding landmark genes (Datasets S5 and S6). The top three highly expressed genes were defined as landmark genes. For HGP of yellow light, molecular chaperone took a great proportion of landmark genes with high expression levels (Figure S12), consistent with the activated protein synthesis in pathways enrichment analysis (Figure 4D) and wet‐lab results (Figure 4E), which further demonstrated the coexpression between nitrate utilization and protein synthesis regulated by yellow light. For blue light, PD genes were assigned to class 3 and class 0 (Figure 5A,B and Dataset S1). Genes that encoded nitrite reductase (*NirK*) and nitrate/nitrite transporter (*NarK1*) could be represented by landmark genes of class 3, including genes that encode myo‐inositol‐1‐phosphate synthase (*MIPS*), nitric oxide reductase subunit B (*NorB*), and isocitrate dehydrogenase (*IDH*). These landmark genes involve in signaling transduction, nitrogen metabolism, and energy production (Text S5), consistent with pathway enrichment analysis and biological prior knowledge of denitrification respiratory chains. Additionally, nitrate reductase (*NarG*) was in class 0 and can be represented by landmark genes that encode superoxide dismutase (*SOD*) and 4‐hydroxy‐tetrahydrodipicolinate synthase (*DapA*), both of which were critical enzymes in antioxidant systems, mainly involved in superoxide scavenging. This was also supported by the domain role of SOD in determining the microbial network in a previous study [35].

As was shown in the topological network models, nitrate reduction mediated by *NarG* was coexpressed with landmark genes that encode enzymes related to superoxide. Therefore, we assayed the superoxide level during photo‐denitrification at different stages (Figure 5C), including nitrate reduction (Stage 1), nitrite reduction (Stage 2), and nitrogen depletion (Stage 3). It turned out that the superoxide level variations under different illumination conditions were consistent with nitrate removal performance in Figure 2A, that is, higher superoxide levels correspond to higher nitrate removal activities. These primarily confirmed the nitrate‐superoxide coregulation predicted by the topological network model. Gradient nitrogen experiments and nitrate presence experiments (Figures 5D and S13) further solidified the coexpression relationship between nitrate reduction and superoxide production.

The divergent trends of total ROS (Figure S10A) and superoxide production (Figure 5C) in response to different illumination conditions implied that superoxide played a pivotal role in light‐regulated nitrate conversion, potentially as signals [36]. Therefore, we conducted quenching experiments under dark, blue, and yellow light to investigate the contributions of typical ROS, including hydroxyl radical (·OH), singlet oxygen (^1^O_2_), and superoxide (·O_2_^−^) (Figure S14B). These results further demonstrated the principal role of superoxide in nitrate removal across all groups, especially for yellow light where superoxide achieved a 99.1% quenching ratio. On the basis of the coexpression relationship between superoxide and nitrate reduction, we developed an enzymatic superoxide generation method to facilitate nitrate removal (Figure S14C,D). It can be observed that after the addition of superoxide at timepoint a, the nitrate removal rate of the test group was boosted (Figure 5E) and achieved a 99.9% higher nitrate removal efficiency at timepoint b compared with the control (Figure 5F).

### The mechanism and potentials of light‐regulated photo‐denitrification

Besides phenotype prediction and regulatory strategies for new biotechnology, the modeling results also enabled mechanistic scheme reconstruction. In the case of photo‐denitrification, these included molecular biology mechanisms for wavelength‐dependent denitrification (Text S6), nitrate‐superoxide coregulation (Text S7), and the wavelength‐divergent secretion system (Text S8) [28].

Overall, the secretion system was the core of cross‐species interaction (Figure 1). Blue light photoreceptors were ubiquitous and have been implemented in a broad spectrum of biological platforms [37, 38], explaining the decentralized metabolism fluxes triggered by blue light in photo‐denitrification. More diverse metabolites were synthesized, typically active substances, like, cofactors and vitamins. Additionally, some of those secreted molecules were crucial for microbiomes to maintain homeostasis under photochemical stress as intercellular signals, which accelerated proliferation and evolution [39]. These metabolites were potential high‐value resources to be recovered, or shed light on new bioprocesses that could utilize nitrate as substrates to save the costs of high‐value chemical production. The superior activation effects of yellow light were intriguing since there were few reports on optogenetic switches of yellow light [37, 38]. This might contribute to the cross‐species interactions. The centralized metabolism fluxes triggered by yellow light were mostly used for protein synthesis, especially pilus‐related proteins, suggesting the role of pilis in accelerating interspecies electron transfer for collective functional metabolisms [40], such as the enriched terpenoids and polyketides in SGPs of yellow light (Figure S11). The novel collective effects of light harbored great potential in bioproduction, developing modules for synthetic biology, and deepening recognitions on biological environmental exposome [41].

## DISCUSSION

**Figure 6:**
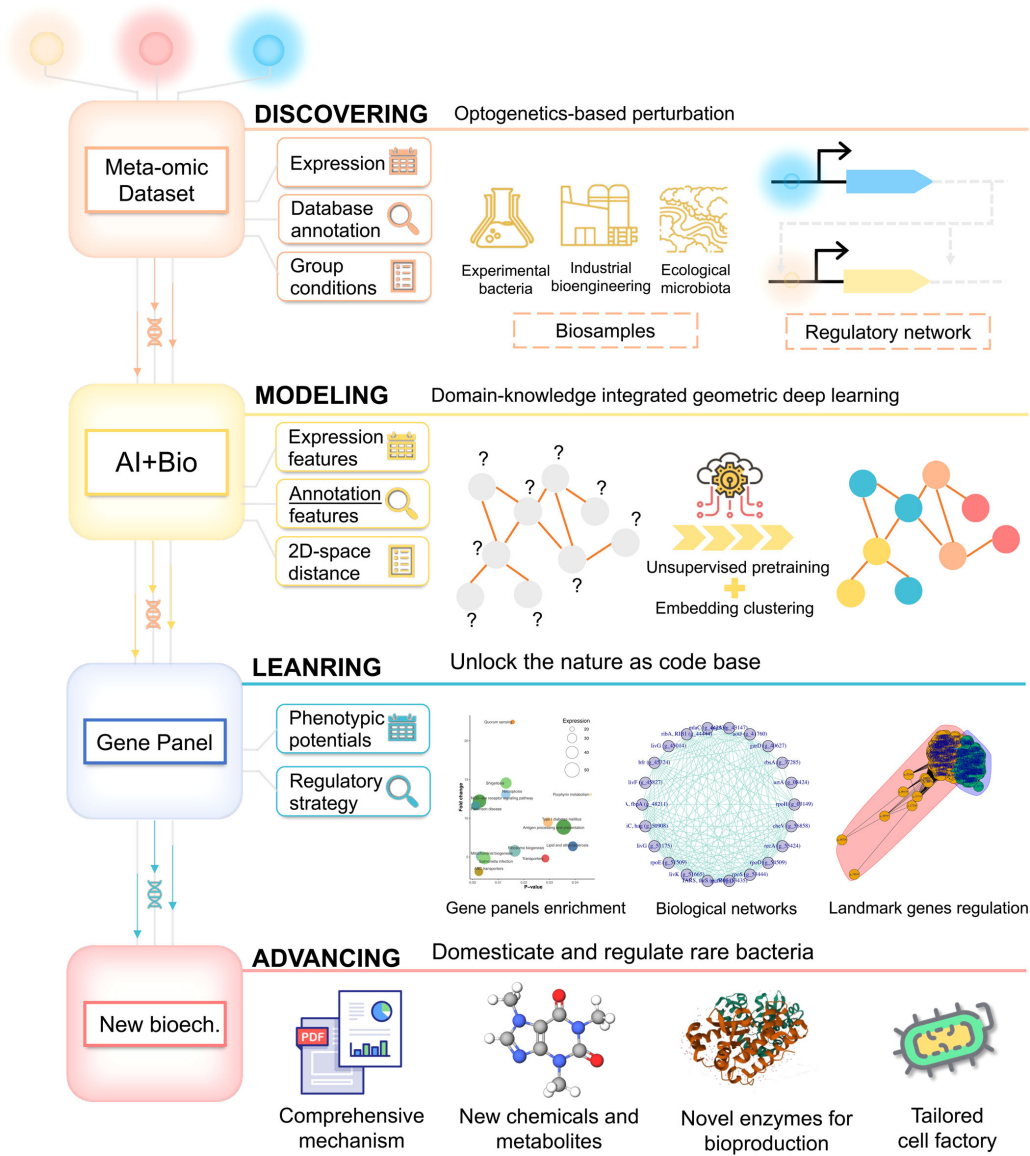
Workflow of the DMLA cycle for unlocking nature‐based advancement. The case scope and theory basis are depicted at the “Discovering” stage. The “Modeling” stage elucidates the core requisites, including geometric deep‐learning models and biological data sets. The “Learning” stage presents the services the app suites could provide. The last stage, “Advancing,” showcases the potential versatile applications of the new biotechnologies derived from the DMLA cycle. 2D, two‐dimensional; DMLA, discover–model–learn–advance.

Unlock nature as a code base for healthy ecosystems, clean energy, and a more sustainable future, which has witnessed the biotechnology boom for the past few years. Though the design‐build‐learn‐test (DBLT) cycle for synthetic biology DBLT exhibited immense potential in accelerating biotechnology advancement [42], most efforts were in model strains, like, *Escherichia coli*, as well as limited to enhancing efficiency and yields. While the decryption of natural microbiomes always remained the bottleneck. The DMLA cycle we showcased here exhibits tremendous potential in unleashing the power of denitrifying microbiomes via optogenetics (Figure 6).

At the “Discovering” stage, environmental perturbation is necessary to activate genetic processes to capture the dynamic regulatory network. Here, we employ optogenetic‐based perturbation, triggering signal transfers through optogenetic switches, the molecular devices for regulating light‐controlled gene expression, protein localization, signal transduction, and protein–protein interactions [38]. Therefore, these methods can also be applied to other microbiomes and meta‐omics besides metatranscriptomics of denitrifiers. These extra demonstrations and guidance are provided at https://github.com/YoungeLiao/DMLA, including simultaneous CO_2_ fixation‐denitrification microbiomes and practical engineering microbiomes (Text S9). The optogenetic‐induced dynamic regulatory network changes are contained in the input data sets, including expression matrix, database annotation, and group conditions. At the “Modeling” stage, the biological topology principles, that is, system biology, are captured by models automatically. Geometric deep‐learning harbors superior capability in characterizing complex topological relationships and integrating heterogeneous information [22, 23]. In addition, compared with other machine learning approaches, the framework could conquer data noises, small sample sizes, and labeling bottlenecks [43, 44]. The biological data sets, including expression levels and subcellular annotations, are engineered as node features and gene expression distance in the 2D latent space as edges. The heterogeneous knowledge is integrated through unsupervised pretraining to obtain embeddings, which are then clustered to obtain coexpressed gene panels. At the “Learning” stage, the app suites unleash the potential of nature's code base. On the basis of the gene panels, enriched pathways unveil unknown phenotypes. The biological network characterized by landmark genes is a powerful tool for elucidating biological mechanisms and developing regulatory strategies. On the basis of these, the new biotechnology could be utilized to domesticate and regulate rare bacteria, providing versatile applications, including comprehensive mechanism reports, new chemicals and metabolites discovery, developing novel enzymes for bioproduction, tailored cell factories for synthetic biology, and so forth.

Previous studies mostly focus on predicting the gene regulatory network in single‐cell data [22, 45], while the natural genetic code library, which harbors enormous potential for sustainable biotechnology, remains greatly natural genetic unknown treasures for mining. Our study showcased how to exploit GNNs to discover new microbial capabilities and develop regulatory strategies on natural microbiomes. This opens up a field for harnessing natural solutions for global challenges, such as nitrogen pollution mitigation, CO_2_ capture, utilization, and so forth. Nonetheless, to make the most of the DMLA cycle to unlock natural potentials, there remain some challenges from both computational and biological perspectives. In the computational aspect, computational power and modeling processes are two major bottlenecks. Meta‐omics usually includes millions of genes, which are hundreds of times higher compared with the biomedical and clinical data sets. Moreover, the traditional methods usually need to manually select suitable model architectures and hyperparameters, which is laborious and costly. Recently proposed automated machine learning [46] and generative model [47] could be promising approaches. In the biological aspect, limited mechanism recognition and lack of gene annotations are challenges for implementing an effective DMLA cycle. More in‐depth recognitions and wet‐lab validations on ecological principles, system biology, and quantum biology are needed, such as quantum coherence in light‐harvesting protein, quorum sensing, and direct interspecies electron transfer in anaerobic digestion [1, 40, 47]. This could also facilitate biological‐tailored modeling to better integrate domain knowledge for effective DMLA cycles. Moreover, besides the light‐wavelength strategy, more regulatory strategies and corresponding data sets are needed to enrich the models to be more intelligent, precise, and universal, which could empower the decryption of natural principles and accelerate the development of biotechnology.

## METHODS

### The optical‐control platform, microbiome, and operation of photo‐denitrification

The light control platform was shielded by tin foil to avoid interference with external light. The LED light band was built into the outer wall of the light control platform to provide a surrounding light field for the reactor in the middle of the platform. The bottom of the light control platform was a magnetic stirrer to ensure full mixing of the reaction. LED lamp beads were embedded on the lamp strip without covering, the lamp bead spacing was 50 mm, the lamp strip width was 8 mm, the rated voltage was 4–6 V, the rated power was 3–5 W/m, and the light intensity of the final space light field was 2.0 ± 0.5 mW cm^−2^. The light wavelength was controlled by using different LED lamp beads as the light source, the light intensity was controlled by the length of the lamp strip, and the uniform distribution of the light field was guaranteed by the symmetrical distribution of the lamp strip lamp beads on the inner wall of the optical‐control platform. The spectra of LED (Figure S1) were characterized by the spectrometer (OceanInsight, Maya2000Pro). The microbiome culturing, activation, and photo‐denitrification were conducted as described in our previous study [10].

### Data preprocessing and exploratory data sets analysis

We obtained DEGs in response to blue and yellow light through DESeq analysis [48]. Genes with log 2 fold change >1 and *p* < 0.05 were regarded as DEGs. Dimension reduction analysis on those genes, including 2D principle component analysis (PCA), 3D‐PCA, and tSNE, were conducted in R. For tSNE analysis, we utilized log normalized expression data (see Equation 1), and set max iteration to 1000, *θ* to 0.4, perplexity to 20, verbose to false. Log normalization and scaling were also performed in the HC.

(1) $$x_{i}=\log_{10}(\mathrm{expre}+1),$$

where *x*_*i*_ was the log normalized gene expression level, *expre* was the gene expression level quantified by FPKM mentioned above.

To decipher the transcriptomics responses of microbiomes through multimodal subdata sets, information that comprises intra‐ and intercellular interaction was critical. Therefore, gene expression information and subcellular location information were input into our model as complementary biological domain knowledge. Gene expression mainly reflected the intracellular regulatory signaling, whereas subcellular location reflects intercellular interaction. To better characterize the topological interaction, we adopted geometric deep‐learning and integrated gene expression information and subcellular location information through unsupervised learning. To alleviate the potential biological data noise in modeling, we filtered low‐expression DEGs with default mean expression <1 to obtain valid DEGs (25,886 in total for metatranscriptomics of photo‐denitrification). Considering denoising, we split the valid DEGs into two data sets, that is, blue light DEGs (25,277 genes) and yellow light DEGs (4366 genes) for modeling. The utilization tutorials and case demonstrations were also given in our code base (https://github.com/YoungeLiao/DMLA) for better comprehension.

### Graph construction

According to the above data preprocessing, the constructed graph data has 25,277 nodes and 4366 nodes for blue and yellow light, respectively, where each node corresponded to a gene and had 8 node features, including 6 columns representing expression information and 2 columns representing subcellular information. The detailed descriptions were as follows.

As for feature engineering, the light‐wavelength‐based expression matrix represented in FPKM was normalized by log10 to obtain nodes features. Subcellular annotations, including signal peptide and transmembrane domain annotations, were engineered as nodes features. We utilized 1 and 0 to represent whether or not peptide and transmembrane protein‐coding genes. The design was based on complex environmental microbiota interaction availability. Genes for proteins with different subcellular locations had different interaction modes. For instance, secretory protein, that is, protein with signal peptide but no transmembrane domain, could have cross‐species impacts, while genes for intracellular proteins were mainly for basic cellular metabolism. The final node features, denoted as X, were the concatenation of gene expression and subcellular location features.

With preprocessed node features, we constructed edges to model the intercellular interaction. We employed 2D tSNE and calculated the Euclidean distances between nodes in the projected 2D plane. We set the maximum iterations of tSNE as 1000 with a perplexity of 20. Then, we built edges between nodes whose distances were smaller than a preset threshold, where the threshold was adjusted so that the average degree of the graph was 5, that is, each gene was connected to the other five genes in expectation. The edges connected to each node were undirected. The intuition is that nodes connected by edges possess similar gene expression patterns regardless of their physical spatial distance. More specifically, owing to the promoter regulatory mechanism or spatial structure of proteins, two genes that are far apart in the sequence can be coexpressed or able to interact with each other [49, 50].

### GNN and unsupervised clustering

On the basis of the constructed graph, we applied GNNs [23], which are state‐of‐the‐art machine learning models for graph data, to learn node embeddings as gene representations and enable further analyses. Specifically, we adopted GCNs [51], the most representative GNNs. One layer of GCNs was calculated as follows:

(2) $$H^{\left(l+1\right)}=\sigma \left({{\tilde{D}}^{-\frac{1}{2}}\tilde{A}\tilde{D}}^{-\frac{1}{2}}H^{\left(l\right)}W^{\left(l\right)}\right),$$

where $H^{\left(l\right)}$ represented the node embedding at the lth layer of GCNs, $\tilde{A}$ represented the adjacency matrix of the graph by adding self‐loops, $\tilde{D}$ represented the diagonal degree matrix: $\tilde{D_{\mathrm{ii}}}=\sum_{j}\tilde{A_{\mathrm{ij}}}$, $W^{\left(l\right)}$ represented the learnable weights, and σ(⋅) was the nonlinear activation function. The node embeddings were initialized as node features in the first layer, that is, $H^{\left(0\right)}=X$. Concretely, the adjacency matrix was calculated as

(3) $$\tilde{A}=\left(1-\alpha \right)A+\alpha I,$$

where I was the identity matrix and α is a hyperparameter to control the strength of self‐loops, which was set as 0.8 in our experiments. Using several layers of GCNs, nodes can exchange information with their neighborhoods and thus learn the intercellular interaction.

Considering the lack of node label information, we adopted DGI [20], a state‐of‐the‐art elf‐supervised algorithm for training GNNs, and based on the modified GNNs [52], to learn node embeddings. Specifically, the objective of DGI was formulated as follows:

(4) $$\begin{array}{ccl} L & = & \sum_{i=1}^{N}E_{A,X}\log\;D\left(h_{i},s\right)\\ & & +\sum_{j=1}^{M}E_{A,X^{\prime }}\log\left(1-D\left(h_{j}^{\prime },s\right)\right), \end{array}$$

where $h_{i}$ was the final node embedding of node i, N was the number of nodes, $h_{j}^{\prime }$ was the embedding of node j in a randomly corrupted graph, M was the number of corrupted samples, s was the summary vector of the graph learned by a readout function to summarize node embeddings, and D was the discriminator. In our experiments, we corrupted the graph by randomly permutating node features, adopted mean pooling as the readout function, and set the discriminator as a bilinear function.

After training and obtaining the node embedding H, we reduced the dimensionality of node embeddings using PCA and used classical vector‐based clustering algorithms to group cells.

We set the number of clusters, denoted as n, based on the denitrification performance in the wet experiment. Typical cluster numbers, including n=24 (eggNOG classification number), 10 and 7 (KEGG level 1 pathways number) were selected to compare their discriminative capability on phototransduction genes. Finally, we used n=7, the best‐performed cluster number for subsequent analyses.

### Model evaluation

We evaluated the genes panel identification capability both qualitatively and quantitatively, as well as validated on biological meanings. HC and *K*‐means, two commonly used clustering methods, were utilized as benchmarks to evaluate the clustering and information integration capability of the DGI model.

For qualitative evaluation, we projected the genes clustering results on 2D space through tSNE and compared the HC, *K*‐means, and DGI methods on the expression matrix, and then further compared the *K*‐means and DGI performance with and without subcellular information. We utilized SCI to quantitatively evaluate the clustering capability of different methods. SCI was used to evaluate the intercluster similarity [53]. We used the DEGs' expression matrix of blue and yellow light groups to represent samples, and the assigned cluster number of genes to represent labels. SCI was calculated based on those representations.

Given the rich information contained in complex environmental microbiota, we extracted high‐credited pathways responsive to light based on literature reviews and other prior knowledge, including phototransduction, light‐sensing pathway; peroxisome and longevity regulating pathway—multiple species, environmental stress‐related pathways; cytochrome P450 and metabolism of xenobiotics by cytochrome P450, pathways that containing blue light receptor. Functional annotations, including Swiss‐Prot and KEGG database, were also included for enrichment analysis and metabolism network reconstruction.

To validate the biological function, we first pictured the expression pattern and cluster assignment of phototransduction. Inspired by the similar expression patterns among light‐responsive pathways, we developed FAS as an indicator to quantitatively evaluate the consistency between clustering results and contextual biological knowledge. *FAS*_*w*_ the FAS of genes assigned to certain pathways *w*, is defined as follows:

(5) $$FAS_{w}=\left\{\begin{array}{ll} \log\;\left(1+\text{exp}\left(\frac{\sum_{i=1}^{n}r_{i}}{r_{w}}\right)\right), & n\leq k,\\ \log\;\left(1+\text{exp}\left(\frac{\sum_{i=1}^{k}r_{i}-\sum_{i=k+1}^{n}r_{i}}{r_{w}}\right)\right), & n>k, \end{array}\right.$$

where n was the total cluster number that genes of the targeted function assigned to. All clusters were sorted by their ratios in the descending order. k was a hyperparameter representing the number of correctly assigned clusters. Considering genes belonging to the same gene panel have either positive or negative effects on the targeted function, we assumed that clusters with the two highest gene ratios were positively clustered genes, that is, set k=2, while the rest were negatively clustered genes. Variable $r_{i}$ was the gene ratio of cluster i to all genes of certain pathway w, which can be calculated as

(6) $$r_{i}=\frac{m_{i}}{N_{w}},$$

where $m_{i}$ was the genes count of cluster i, and $N_{w}$ was the total number of genes assigned to pathway w.

Variable $r_{w}$ denoted the genes ratio of pathway *w* to all DEGs:

(7) $$r_{w}=\frac{\sum_{i=1}^{n}m_{i}}{M_{g}},$$

where $M_{g}$ was the total gene counts of data sets.

### Spatial distribution and pathway enrichment analysis on photo‐denitrification genes

To elucidate the mechanism of light‐induced responses, we extracted the major clusters that nitrogen metabolism and phototransduction genes were subjected to (Figure S8), that is, clusters 7 and 4 for blue light and clusters 6, 3, and 4 for yellow light. We extracted highly expressed genes related to nitrate and nitrite metabolism (Figure S6B) to represent key genes in PD activity. Specifically, nitrate‐ and nitrite‐related genes, that is, PD genes, were extracted by keywords based on the Swiss‐Prot description. We chose the Swiss‐Prot database for its more complete annotations than other databases. Given the lowly expressed genes had negligible effects on collective behaviors of microbiomes, we filtered low‐expression genes with a mean expression of less than 1. After searching in Swiss‐Prot and filtering low‐expression genes, we merged the eligible genes of blue and yellow light to obtain the PD gene set.

We projected the expression spatial distribution of these functional genes in the latent space through tSNE to depict the overall expression pattern. To identify essential pathways in functional genes panels, we annotated these genes to KEGG level 3 pathways and summarized the expression pattern in response to blue and yellow light, respectively. To identify the major pathways corresponding to the phenotype for microbial collective behavior prediction, it was necessary to filter low‐expression and nonsignificant pathways. For blue light, pathways with *p* < 0.01, fold change <0.5 or >2, and expression level <10 FPKM were selected as significant photo‐denitrification pathways. Similarly for yellow light, and finally obtained top significant pathways that meet the filtering requirements. Fold changes were calculated with the dark group as control.

### ROS detection and analysis

1,3‐Diphenylisobenzofuran (DPBF), a ROS fluorescence probe, was employed to evaluate total ROS production by quantifying the DPBF consumption [54]. Briefly, DPBF was dissolved in 75% ethanol and 2.5 mM DPBF was added to the 96‐well plates. Ultrapure water was used to control the total assaying volume. After the addition of the cell sample, the ultraviolet–visible (UV–Vis) absorption spectrum was continuously monitored by a microplate reader (Thermo Fisher Scientific, TENCAN‐Spark) for 30 min with an interval of 10 s. On the basis of the UV–Vis absorbance spectra (Figure S14A), Abs_410_ was chosen to monitor the total ROS production. To derive the kinetic constant of total ROS production under different light, timepoints with sufficient substrates (Abs_410_ > 0.85) were utilized to calculate the DPBF consumption rate, that is, the total ROS production rate. The mathematical equation is as follows:

(8) $$k_{\mathrm{ROSt}}=\frac{1-\frac{A_{t}}{A_{0}}}{{\mathrm{OD}}_{600}}\times 10^{3},$$

where *k*_*ROSt*_ was the total ROS production rate at time *t*, *A*_*t*_ and *A*_0_ were 410 nm absorbance at time *t* and initial time. *OD*_600_ was utilized to represent cell density for normalization. The 10^3^ was utilized to adjust calculation results to a suitable range for comparison.

### Extraction of EPS and self‐catalysis experiments

After photo‐denitrification, EPS was extracted following the methods reported in the previous study with the appropriate modification [55]. Briefly, cell suspensions were shocked well and centrifugated at 4000*g* for 15 min at a temperature of 4°C. The supernatant was collected as soluble EPS (S‐EPS). Further, the residual cells were resuspended in 5% NaCl solution and agitated by vortex mixer (Scientific Industries, Vortex Genie2) for 3 min, followed by water bath at 60°C for 3 min, and agitated again for 3 min. The mixture was then centrifugated for 15 min at 4000*g* and 4°C, the obtained supernatant was collected as loosely bound EPS (LB‐EPS). To obtain the tightly bound EPS (TB‐EPS), the remained sludge was resuspended in 5% NaCl solution and rapidly agitated for 3 min as mentioned above followed by a 30 min water bath at 60°C, and then agitated again for 3 min. After that, the mixture was centrifugated for 15 min at 10,000*g* and 4°C, and the supernatant was collected as TB‐EPS. Equal S‐EPS, LB‐EPS, and TB‐EPS were mixed together as mixed EPS (M‐EPS). M‐EPS scavenged from different illumination groups were utilized as biocatalytics to facilitate denitrification of model denitrifier, *Paraccocus denitrificans* (*P. denitrificans*) under dark conditions. Equal amounts of M‐EPS were added at the start of denitrification, and nitrate removal efficiency was quantified after about 24 h.

### Gene topological network construction and landmark gene identification

We constructed gene topological networks for HGP and SGP. For blue light, genes assigned to cluster 4 and cluster 5 were extracted as the HGP and SGP, respectively. For yellow light, correspondingly cluster 3 and cluster 1. Genes assigned to phototransduction were also extracted and integrated into the topological network. We constructed the networks in Gephi 0.10.1. Pearson correlation coefficients were adopted to obtain the correlation matrix. To obtain high‐credit edges, edges with correlation coefficients <0.9 and *p* > 0.05 were filtered for blue light. Degree, weighted degree, modularity class, eccentricity, closeness centrality, harmonic closeness, betweenness centrality, and clustering coefficient were obtained through the build‐in algorithm of Gephi 0.10.1. The results can be found in Datasets S1–S4. Mean expression levels of samples exposed to light were utilized to distinguish landmark genes. The top three genes among the modularity class were defined as landmark genes of this class.

### Superoxide detection and analysis

Extracellular production of superoxide was evaluated by MCLA, a chemiluminescence probe [55]. Chemiluminescence elicited by the reaction of MCLA with superoxide or the singlet excited state of dioxygen was monitored by a microplate reader (Thermo Fisher Scientific, TENCAN‐Spark) for 30 min. An additional control group with SOD as the superoxide scavenger was set for each sample. After adding MCLA and SOD, cell suspensions extracted by syringes from photo‐denitrification reactors were added and monitored in the microplate immediately. The chemiluminescence difference of dynamically stable points was employed to gunge the superoxide level. To alleviate the impact of cell density, we adjusted cell density to a similar level and normalized the chemiluminescence difference with *OD*_600_ as well.

### Biological method to supplement superoxide

We employed a mild biological method to add superoxide during denitrification. First, we set triplicate test and control groups, performing denitrification under dark conditions without the addition of superoxide to ensure these two groups had similar capabilities in denitrification. After most of the nitrate was removed (at about 21 h), we supplemented superoxide and nitrate at the same time, and monitored the nitrate concentration continuously. The superoxide supplemented in the denitrification system was generated by xanthine oxidation catalyzed by xanthine oxidase. We validated the methods through the superoxide detection experiment (Figure S14C) and the nonbiological nitrate‐superoxide experiment (Figure S14D). All the regents, including xanthine, xanthine oxidase, and water‐soluble tetrazolium 8 were purchased from Dojindo Laboratories. In the superoxide detection experiment, superoxide was generated by xanthine oxidation following the instructions. No cells were added to all groups. Peak absorbance at about 450 nm can only be detected at the superoxide group without the addition of SOD (10 kU), which demonstrated the feasibility of this method to generate superoxide and SOD as the superoxide scavenger. Samples of nonbiological experiments were collected after about a day to assay the nitrate concentration. It was evident that nitrate cannot be removed by superoxide (Figure S14D).

## AUTHOR CONTRIBUTIONS

Yang Liao did the experiments, analyzed data, and wrote the manuscript. Jing Zhao and Jiyong Bian did the experiments and revised the manuscript. Ziwei Zhang analyzed the data and revised the manuscript. Siqi Xu and Rui Li did the experiments. Yijian Qin analyzed data. Shiyu Miao and Meng Zhang provided materials and equipment. Ruiping Liu, Wenwu Zhu, Huijuan Liu, and Jiuhui Qu supervised this project.

## CONFLICT OF INTEREST STATEMENT

The authors declare no conflict of interest.

## Supporting information


**Figure S1**: The Light spectrums of LEDs employed to regulate photo‐denitrification.
**Figure S2**: 3D Fluorescence images that depicted the cellular viability and membrane damage.
**Figure S3**: Gene expression profiles and dimension reduction analysis.
**Figure S4**: EggNOG class distribution on all differentially expressed genes (DEGs).
**Figure S5**: Subcellular location profiles of blue light's and yellow light's valid DEGs.
**Figure S6**: Expression patterns of major DEGs that related to phototransduction and nitrate conversion.
**Figure S7**: Cluster assignment of phototransduction genes under different cluster number.
**Figure S8**: Cluster distribution of functional pathways.
**Figure S9**: Pathway enrichment analysis on significant photo‐denitrification pathways of blue and yellow light.
**Figure S10**: Wet‐lab validations on co‐expression between total reactive oxygen species (ROS) levels and photo‐denitrification.
**Figure S11**: Pathways enrichment analysis of signaling gene panels (SGPs) of blue and yellow light.
**Figure S12**: The topological network model and corresponding landmark genes of yellow light's HGP.
**Figure S13**: Co‐expression of nitrate metabolism and superoxide production.
**Figure S14**: ROS assay and addition of superoxide.
**Figure S15**: Mechanistic scheme of light‐regulated denitrification.

**Table S1**: Statistic summary of unigenes obtained through clean reads assembly.
**Table S2**: Highly expressed genes that encode phototransduction of blue and yellow light datasets.
**Table S3**: Annotations of nitrate‐ and nitrite‐related genes, i.e. partial denitrification genes (PDGs).
**Table S4**: Pathways enrichment analysis on the HGPs and SGPs of blue and yellow light.
**Table S5**: Topological properties of HGPs and SGPs.

## Data Availability

All the sequencing data were deposited in the China National Center for Bioinformation database under BioProject PRJCA017836 (https://ngdc.cncb.ac.cn/search/?dbId=&q=PRJCA017836) and National Center for Biotechnology Information under BioProject PRJNA984758 (https://www.ncbi.nlm.nih.gov/bioproject/PRJNA984758). Other data and codes related to the study are included in the article and/or supporting information. Codes are available on GitHub (https://github.com/YoungeLiao/DMLA). Supplementary materials (figures, tables, scripts, graphical abstract, slides, videos, Chinese translated version, and update materials) may be found in the online DOI or iMeta Science http://www.imeta.science/.
