# Supplementary material for: From mechanism to application: Decrypting light‐regulated denitrifying microbiome through geometric deep learning
Source: Imeta. 2024 Jan 6;3(1):e162. doi: 10.1002/imt2.162 (PMC10989148; doi:10.1002/imt2.162)
Supplement: Supplementary file 1 — Figure S1: The Light spectrums of LEDs employed to regulate photo‐denitrification. Figure S2: 3D Fluorescence images that depicted the cellular viability and membrane damage. Figure S3: Gene expression profiles and dimension reduction analysis. Figure S4: EggNOG class distribution on all differentially expressed genes (DEGs). Figure S5: Subcellular location profiles of blue light's and yellow light's valid DEGs. Figure S6: Expression patterns of major DEGs that related to phototransduction and nitrate conversion. Figure S7: Cluster assignment of phototransduction genes under different cluster number. Figure S8: Cluster distribution of functional pathways. Figure S9: Pathway enrichment analysis on significant photo‐denitrification pathways of blue and yellow light. Figure S10: Wet‐lab validations on co‐expression between total reactive oxygen species (ROS) levels and photo‐denitrification. Figure S11: Pathways enrichment analysis of signaling gene panels (SGPs) of blue and yellow light. Figure S12: The topological network model and corresponding landmark genes of yellow light's HGP. Figure S13: Co‐expression of nitrate metabolism and superoxide production. Figure S14: ROS assay and addition of superoxide. Figure S15: Mechanistic scheme of light‐regulated denitrification. [file IMT2-3-e162-s002.docx]

**Supporting Information**

**From mechanism to application: decrypting light-regulated denitrifying microbiome through geometric deep learning**

Yang Liao^1^, Jing Zhao^1^, Jiyong Bian^1^, Ziwei Zhang^2^, Siqi Xu^a^, Yijian Qin^2^, Shiyu Miao^1^, Rui Li^1^, Ruiping Liu^1^*, Meng Zhang^3^, Wenwu Zhu^2^, Huijuan Liu^1^, Jiuhui Qu^1^.

^1^ Center for Water and Ecology, State Key Joint Laboratory of Environment Simulation and Pollution Control, School of Environment, Tsinghua University, Beijing 100084, China

^2^ Department of Computer Science and Technology, Tsinghua University, Beijing 100084, China;

^3^ School of Electronic and Information Engineering, Beihang University, Beijing, 100191, China

*Correspondence: [rpliu@tsinghua.edu.cn](mailto:rpliu@tsinghua.edu.cn) (Ruiping Liu)

**This file includes:**

Supplementary Methods

Supplementary Texts

Figures S1 to S15

Legends for Datasets S1 to S7

Supplementary References

**Other supporting materials for this manuscript include the following:**

﻿ Table S1 to S5, Source Data

Datasets S1 to S7

Supporting Information

Supplementary Methods

## Fluorescence assay. Bacterial Viability Detection Kit (DOJINDO) was used for cell staining. Briefly, before staining, the cell density of bacterial suspension was adjusted to similar level and washed by PBS (0.01 M, pH = 7.4) twice. After that, we used CFDA, a coloring reagent that can fluoresce when binding to intracellular esterase, indicating esterase activity and cellular viability. PI, a coloring reagent that can combine with nucleic acid, was utilized to indicate dead cells. Stains and cell suspension were vortexed to mix well and follow the instruction for further sample pretreatment. The fluorescence images of stained cells were shotted by confocal laser scanning microscopy (CLSM, Zeiss LSM880). The CLSM data were further processed by ZEN 3.1 (blue edition) to obtain 2.5D images to enhance the resolving power of cellular physiology after photo-denitrification under different illumination conditions.

## RNA extraction and metatranscriptomic sequencing. After photo-denitrification, cell suspensions were collected for RNA extraction. Total RNAs were extracted with the E.Z.N.A.® Soil RNA Midi Kit (Omega Bio-tek, Norcross, GA, U.S.) following the manufacturer’s instruction. The RNA concentration and purity were quantified with NanoDrop2000 (Thermo Fisher Scientific,U.S.). RNA quality assessment was conducter with a RNA6000 Nano chip (total RNA) in an Agilent 2100 Bioanalyzer, and further determined by RNA integrity number (RIN). Ribo-zero Magnetic kit was used for rRNA removal followed the manufacturer’s instruction (Epicentre, an Illumina® company). cDNA libraries were constructed using TruSeq™ RNA sample prep kit (Illumina). The barcoded libraries were paired-end sequenced on the Illumina Hiseq 2500 platform at Majorbio Bio-Pharm Technology Co., Ltd. (Shanghai, China), using HiSeq 4000 PE Cluster Kit and HiSeq 4000 SBS Kits.

## Metatranscriptomic data quality control and genes expression quantification. To obtain high-quality reads, SeqPrep (<https://github>.com/jstjohn/SeqPrep) and Sickle (<https://github>.com/najoshi/sickle) were utilized to strip 3’ and 5’ ends and remove low-quality reads (length < 50 bp or quality value < 20 or having N bases), respectively. rRNA reads were removed using SortMeRNA [1] aligning to the SILVA 128 version database. Clean reads were assembled by Trinity [2] and the longest transcripts were extracted through python scripts as unigenes. Then, open reading frames (ORFs) prediction were performed with TransGeneScan [3] and sequences with 95% identity were clustered as the non-redundant gene catalogues by CD-HIT [4]. The longest sequence was utilized to represent the gene catalogue. After that, reads were mapped to representative genes and gene expression was quantified in the form of Fragments Per Kilobase of transcript per Million fragments mapped (FPKM) that calculated by RSEM [5] to eliminate the disturbance caused by genes length and sequencing depth.

## Gene functional annotations. Taxonomic annotations were carried out through aligning non-redundant gene catalogs against NR database with by BLAST [6]. Furthermore, genes were aligned against eggNOG (evolutionary genealogy of genes: Non-supervised Orthologous Groups) and KEGG database (Kyoto Encyclopedia of Genes and Genomes) to obtain functional information via Diamond (http://www.diamondsearch.org/index.php, version 0.8.35). The subcellular information, including signal peptide and transmembrane domain, were predicted using Diamond against SignalIP 6.0 database (https://services.healthtech.dtu.dk/service.php?SignalP) and TMHMM/2.0 database (http://www.cbs.dtu.dk/services/TMHMM/), respectively. All the e-value cutoff was set as 1e^-5^.

## Spatial distribution and enrichment analysis on significant functional pathways. We conducted enrichment and function analysis based on KEGG database on critical clusters and functionally co-expressed genes. We projected the distribution of critical clusters on 2D tSNE space to decipher the overall expression pattern. To identify essential pathways in functional genes panels, we annotated these genes to KEGG level 3 pathways and summarized the expression pattern in response to blue and yellow light. For blue light, pathways with p-value < 0.01, fold change < 0.5 or > 2 and expression level < 10 FPKM were selected as significant functional pathways. For yellow light, we adjusted the threshold of p-value and expression level to select about top 10 significant pathways.

## Demonstrating the effects of nitrate on superoxide level. Gradient nitrate concentrations (0, 100, 500, 1000 mg N L^-1^) batches were employed to investigate the effects of nitrate concentration on cellular total ROS production and superoxide level. After activation, microbiota were incubated with and without nitrate for 15-20 hours, at the end of which most of the nitrate was depleted due to bio-denitrification under yellow and dark conditions. All gradient experiments were performed under dark conditions to control nitrate to be the only variable, thus proving nitrate reduction, superoxide production and some light-sensing genes were in a co-expression genes module. Photo-denitrification with and without nitrate under yellow and blue light conditions was carried out to investigate the “bio-quenching” effects of nitrate on cellular superoxide. The initial nitrate concentration was 500 mg NO_3_-N L^-1^. Detailed implementations were similar to the gradient experiments as mentioned above.

## Quenching experiments. To investigate the contribution of superoxide on nitrate removal, we conducted quenching experiments on nitrate under dark, blue and yellow light. The variation of nitrate concentration with different illumination conditions after incubation with quenching agents was used to quantify the quenching ratio of different ROS. Dimethyl sulfoxide (DMSO) was employed as ·OH scavenger, and L-histidine for ^1^O_2_, SOD for ·O_2-_, respectively. 1 mM of quenching regents were supplemented to reactors at the start of photo-denitrification. After about 14 hours, nitrate concentrations were measured to calculate quenching ratios. Quenching ratio (QR) was calculated as follow:

$A=\frac{\frac{c_{N0}-c_{\mathrm{Nt}}}{c_{N0}}-\frac{c_{q0}-c_{\mathrm{qit}}}{c_{q0}}}{\frac{c_{N0}-c_{\mathrm{Nt}}}{c_{N0}}}\times100\%$, (1)

where c_N0_ (mg NO_3_-N L^-1^) and c_q0_ (mg NO_3_-N L^-1^) were the initial nitrate concentration of control and quenching group, c_Nt_ (mg NO_3_-N L^-1^) and c_qit_ (mg NO_3_-N L^-1^) were the nitrate concentration of control and quenching group during nitrate reduction.

## Analytics. During photo-denitrification, cell suspensions were periodically sampled by syringe for further assays. After filtered by 0.22 μm micropore filter, nitrate, nitrite and acetate concentration ^5,39^were assayed through iron chromatography (Thermo Fisher Scientific, Integrion ICS-5000). All UV-vis spectra and absorbance were obtained through microplate reader (Thermo Fisher Scientific, TENCAN-Spark). Protein concentrations were quantified by the Micro BCA Protein Assay Kit (Thermo Fisher Scientific Inc., USA).

## Statistics and visualization. All statistical analysis and bioinformatics visualization were performed through R (4.2.2). The results of experiments were shown as means ± standard deviation. Unsupervised learning and model evaluation were conducted through Python. Data visualization was performed in R. Some elements and components for schematic illustrating were downloaded from Servier Medical Art (https://smart.servier.com/), licensed under a Creative Commons Attribution 3.0 Unported License.

Supplementary Texts

## Text S1. Exploratory analysis on datasets. Light irradiation activated transcriptional activities, increasing transcripts number by 137.4% for blue light and 17.9% for yellow light (Table S1). Compared to yellow light, blue light triggered a higher number of differentially expressed genes (DEGs), as well as more substantial and significant transcriptional variance with higher p-value (Figures S3A and 3B). Among 56,991 genes, blue light induces more differentially expressed genes (DEGs) (25,277 genes) than yellow light (4366 genes), and more genes were up-regulated after light illumination, indicating that blue light triggered metabolism fluxes redirection to some rare metabolism pathways. This could be attributed to the activation of genetic parts or components, such as promoters, ion channels, and pumps [7]. This was also supported by the volcano plots (Figure S3B) that blue light displayed notable transcriptional changes and most of the top 10 highly-expressed genes were down-regulated. Whereas most of the highly-expressed genes of yellow groups remained minor changes.

Dimension reduction on samples under different light exposure conditions revealed the light-induced phenotype divergence (Figure S3C). The expression profiles induced by blue light clustered far apart from dark and yellow light groups in both 2D and 3D PCA. Yellow light groups exhibited similar PC1 and PC2 with dark, which possessed cumulative 64.6% and 94.3% of variance. Whereas divergence presented in PC3 which accounts for 2.9% variance, implying that yellow light also induced slight differences in the whole gene expression profile. Dimension reduction on genes through T-distributed stochastic neighbor embedding (tSNE) depicted the spatial distribution of valid DEGs and the light-dependent clustering was evident, indicating the light-induced genetic co-expression (Figure 2B).

Light-induced metabolism changes can be attributed to gene co-expression, i.e. expression levels of photo-responsive genes could impact the expression of genes in the same gene panel through the gene regulatory network [8, 9]. Orthologous groups and functional annotations of database are commonly used to define co-expressed gene panels [10]. We annotated the valid genes to eggNOG database and labeled the eggNOG classification in tSNE space. No obvious gene cluster was formed, indicating that eggNOG failed to capture the light-induced co-expression (Figure S4). Moreover, the lack of annotation, denoted by NA, was another bottleneck in decrypting the optogenetic regulatory mechanism. The above results showed that the prior-knowledge-based methods cannot apply to all situations. Therefore, contextually customed models were needed [11].

We made subcellular information annotations on all genes as described in Material and Methods to supplement the biological knowledge for modeling. There were 1285 genes predicted to encode secretory protein (Figure S5A). Blue light contributed to a larger proportion of genes (4.93% DEGs) that encoded secretory protein compared to yellow light, whereas yellow light triggered the expression of transmembrane protein (Figure S5B).

## Text S2. Determination of gene panels and results evaluation. To better define co-expression gene panels in accordance with biological meaning, we need to set appropriate cluster number. Too large cluster number might separate co-expressed functions into sub-clusters, while too small value might bring about multi-functions in one cluster. We compared DGI and K-means with and without subcellular information to determine the number of co-expressed gene panels (Figures S7, 3C, and 3D). Hierarchical clustering (HC) was not taken into consideration due to its severe bias (Figures 3A and 3B). We compared cluster number of 7, 10 and 24. It can be observed that DGI model performed better than K-means regardless of cluster number. We set cluster number as 7 for further analysis given the better distinguished capability as regard to both clustering approaches and heterogenous information integration.

The Silhouette Coefficient Index (SCI), an inter-cluster similarity indicator, dropped dramatically when combining expression matrix and subcellular information. For blue light, the integration of subcellular information significantly improved the clustering performance. While yellow light presented no significant improvement, which could be attributed to less pronounced expression variance and smaller dataset size [12].

We defined functional assignment score (FAS) to evaluate the biological function matching level quantitatively. We compared the FAS of pathways closely related to light, including oxidative stress and optogenetic switches. Longevity regulating pathway and peroxisome involve in oxidative stress resilience. Cytochrome P450 (CYP450) is responsive to light-wavelength at 450 nm. Metabolism of xenobiotics by Cytochrome P450 and peroxisome exhibited more effective enhancement by DGI model and integration of subcellular information. Xenobiotics were chemicals that involve in the inter-species signaling and substance exchanges [13]. Generally, DGI model outperformed K-means and the integration of subcellular information assisted identify biological functions (Figure 2E). FAS of yellow light groups were higher than blue light, which was attributed to the smaller size of the yellow light dataset so that genes tended to co-express. With the increase of gene counts, pathways would contain more hub gene nodes in metabolism network that involve multi-functions with divergent expression patterns. Nitrogen metabolism was a good case in point [14]. The various expression patterns well supported it (Figure S6B). The divergent performance of blue and yellow light was attributed to the size of datasets that larger datasets contain more hub genes and pathways that involve multi-functions with divergent expression patterns, such as nitrogen metabolism [14]. The various expression patterns well supported it (Figure S6B).

## Text S3. Detailed functions analysis on highly-expressed pathways of HGPs in enrichment analysis. Aging was shared by the HGPs of both blue and yellow light (Figures 3C and 3D). Aging in bacteria refers to the process of senescence, where microorganisms undergo a decline in viability and fitness over time. This could be attributed to the accumulation of cellular damage over time, such as DNA damage, oxidative stress, and protein misfolding, as well as regulated by genetic and environmental factors like nutrient availability, stress responses, and quorum sensing [15]. The representative pathway in KEGG Brite Aging is the longevity regulating pathway, a set of genes associated with signaling, oxidative stress, and protein synthesis that contribute to the collective response to promote cellular fitness and ultimately longevity.

For other pathways in blue light’s HGP, signaling transduction, another highly-expressed KEGG Brite, facilitated the signaling process needed for aging. Specifically, FoxO signaling pathway includes genes that involve in the regulatory network of longevity, oxidative stress resistance, and cell-cycle control, so it is also relevant to ROS [16]. MAPK signaling pathway is another signaling transduction pathway that was essential in cell signaling, proliferation, growth, and division [17]. Besides ROS-related pathways, the expression levels of Inositol phosphate metabolism, and streptomycin biosynthesis were also striking. The inositol phosphate signaling network is central to nutrient responses and coordinates cellular responses to nutrient uptake and utilization from growth factor signaling to energy homeostasis [18]. Moreover, the presence of myo-inositol can promote the biosynthesis of streptomycin as an intermediate [19]. Streptomycin is a broad-spectrum antibiotic, mainly produced by Streptomyces griseus to compete with other bacteria [20]. Therefore, those two pathways play pivotal roles in bacterial virulence, proliferation, apoptosis, and cross-species competition.

As for other pathways in HGPs, blue light was featured with more pathways that involved in ROS production and cellular antioxidant capability than yellow light. Generally, most of disease-related pathways involve in the generation of ROS, which explained the presence of Chemical carcinogenesis – reactive oxygen species, Lipid and atherosclerosis, Pertussis, Legionellosis, and Shigellosis for blue light, Tuberculosis for yellow light. Corresponding to the ROS-related pathways in HGPs of blue light, there were also large number of antioxidant pathways. Peroxisome is the principal pathway in the antioxidant system, containing a set of antioxidant enzymes that are vital in converting ROS into safer molecules. Glutamatergic synapse and GABAergic synapse involved in glutathione metabolism, which was a critical antioxidant compound.

In contrast, the rest pathways of yellow light’s HGP were mostly for protein synthesis, such as chaperone and folding catalysts, exosome, messenger RNA biogenesis and secretion system. Exosomes are small extracellular vesicles secreted and played a role in intercellular communication, such as transmitting signals and molecules and remodeling the extracellular matrix [21], capable of carrying a variety of cargos like proteins, lipids, and genetic materials [22]. The significant enrichment and expression levels of exosomes and Chaperones and folding catalysts, along with secretion system, suggested that the yellow light triggered highly-active proteins secretion for microbial interactions. The activated glyoxylate and dicarboxylate metabolism corresponded to the activated acetate utilization (Figure 1A). The topological properties of four gene panels analyzed above diverged (Table S5).

## Text S4. Detailed functions analysis on highly-expressed pathways of SGPs in enrichment analysis. Among the SGP of blue light (Figure S11A), Carbapenem biosynthesis, porphyrin metabolism, and sulfur relay system were dominant pathways with relatively high expression (Table S4). Porphyrin metabolism involves intermediate metabolites in the production of vital molecules such as heme, chlorophyll coenzyme F430, cobalamin (vitamin B12 coenzyme). These substances were crucial cofactors for basic cellular metabolism. Folate (vitamin B9) is also one of them, explaining the upregulation of folate biosynthesis. Correspondingly, sulfur relay system was also upregulated to facilitate the biosynthesis of those cofactors through signaling messengers like Ubiquitin and ubiquitin-like proteins (Ubls). Additionally, both sulfur relay system and carbapenem biosynthesis play essential roles in protein synthesis, folding and modification. Besides those dominant pathways, other pathways were mostly related to inter-cellular interactions, such as signaling, substances exchanges, and aggregations. Terpenoid is a group of active substances with diverse biological functions, whose mostly well-known application is medicine. Galactose metabolism is essential for the formation of biofilm, which contained exopolysaccharides (EPS) and can assist bacteria to be more resistant to antibiotics like streptomycin [23], corresponding to the presence of Exopolysaccharides biosynthesis, streptomycin biosynthesis. This was because blue light caused stress on microbiota and only species capable of secreting EPS for protection and producing active or antibacterial substances to compete with other species could survive [24, 25]. Correspondingly, secretion system of blue light was also presented in SGP, playing a role in cellular protection, inter-cellular signaling, and substance exchange. To sum up, most of SGP’s pathways of blue light were associated with inter-species signaling for survival under environmental stimulation.

As for yellow light, sulfur metabolism exhibited strikingly high enrichment (Figure S11B), contributing to energy metabolism of phototrophic organisms [26]. In contrast, the expression level of yellow light’s SGP was significantly lower compared to blue light, and ROS-related pathways took the majority, including Chemical carcinogenesis – reactive oxygen species, Cardiac muscle contraction and ascorbate and aldarate metabolism. Ascorbate and aldarate metabolism is a biochemical pathway involved in the breakdown and synthesis of ascorbic acid (vitamin C) and related compounds. These compounds play a crucial role in antioxidant capability. It is reported that exposure to light can increase the ascorbate level [27]. The activated ROS-related pathways implied that light-induced ROS might serve as signals or facilitate signal transduction to regulate diverse microbial metabolism [28]. Other two pathways with relatively high expression level were insect hormone biosynthesis and Limonene and pinene degradation, which involve signaling related to the metabolism of fatty acids, isoprenoids and cytochrome P450 (CYP15A1) [29, 30]. The rest significant pathways with high fold changes and considerable expression levels were mainly related to xenobiotics and amino acid metabolism, i.e. Chloroalkane and chloroalkene degradation, Phenylalanine, tyrosine and tryptophan biosynthesis, etc. Chloroalkane and chloroalkene degradation is the second largest xenobiotic biodegradation pathway [31]. This indicated that yellow light might promote the synthesis of macromolecules like secretory proteins, corresponding to the up-regulated protein synthesis activities in HGP (Figure 4D). The topological analysis of all the gene panels mentioned further support the results. The HGP of yellow light displayed an evident smaller scale compared to blue light’s that only 14 hub genes in total for yellow light whereas 64 hub genes for blue light (Datasets S1 and S2). Additionally, most of closely-correlated hub genes of yellow light were related to protein synthesis (Figure S12), mostly pilus assembly proteins and molecular chaperones. Combining with the up-regulated protein synthesis in pathways enrichment analysis, it could be inferred that yellow light centralized the metabolism fluxes to synthesize proteins, especially pilus, which can be utilized to accelerate electron transfer and energy metabolism [32]. In contrast, blue light possessed a larger number of hub genes (Figure 5A), corresponding to the metabolism profiles in Figure 2A and decentralized metabolism fluxes from HGP to SGP as mentioned above.

## Text S5. Landmark genes analysis distinguished high-resolution co-expression for regulation strategy development. We defined landmark genes as the top three highly-expressed genes among the modularity class. We searched the modularity classes, including narG (g_51049, P09152, nitrate reductase), narK1 (g_19066, Q9RA46, nitrate/nitrite transporter) and nirK (g_06597, P25006, nitrite reductase), and conducted subnetwork co-expression analysis as follows.

For blue light, both narK1, and nirK belonged to class 0, whose landmark genes were Myo-inositol-1-phosphate synthase (MIPS), nitric oxide reductase subunit B (NorB) and isocitrate dehydrogenase (IDH) (Dataset 5, Figure 5*b*). MIPS is an important enzyme involved in the biosynthesis of phosphatidylinositol and phosphoinositides, and plays important roles in signal transduction, cellular growth, etc [33]. NorB was the enzyme for next step of denitrification after nitrite reductase and co-expressed with narK. IDH plays a crucial role in tricarboxylic acid cycle (TCA cycle), which is essential in energy production and cellular metabolism. Therefore, nitrite reduction and its transport are highly associated with the energy supply and can be induced by some active substances like inositol. Nitrate reductase narG belonged to class 3, characterized by superoxide dismutase (SOD), ribosomal protein, and 4-hydroxy-tetrahydrodipicolinate synthase (DapA). DapA involves in lysine biosynthesis, which is also a precursor to many proteins and serves as a building block for glutamate, playing a role in cellular antioxidant capability [34]. Superoxide dismutase was the top landmark gene, as well as vital in ROS scavenging and signaling, indicating superoxide’s association with denitrification.

For partial denitrification (PD) and phototransduction genes of yellow light datasets, only narK1 presented in HGP but without any connection with other nodes (Figure S12). The expression levels of molecular chaperone, the landmark genes of yellow light’s HGP, were striking (Dataset S6), indicating that yellow light centralized the metabolism fluxes for protein synthesis. LuxR family is a DNA-binding transcriptional regulator that can regulate the expression of plenty of crucial physiological events, such as virulence factor production, biofilm formation, quorum sensing (QS), acetate metabolism, motility, bioluminescence, etc. [35, 36] It also presented in the landmark genes of yellow light’s HGP (Dataset S6), inferring its role in sensing light signals. Pilus synthesis was also considerable, indicating it played a role under yellow light illumination. Combining with the overall profiles of yellow lights’ landmark genes of HGP and functional pathways analysis in Figure 4D, it could be learned that yellow light promoted ROS generation to signal protein synthesis and secretion.

Regarding to SGPs, some landmark genes in SGP of blue light also involved in the synthesis of vital antioxidants and enzymes related to oxidative stress release (Dataset S5). A large number of landmark genes involved in the metabolism of antioxidants, such as peroxiredoxin, alkyl hydroperoxide reductase and dye decolorizing peroxidase. These enzymes can catalyze reactions that produce or mediated typical antioxidants, including vitamin C, vitamin E, glutathione (GSH) and flavonoids [37, 38]. In contrast, most of landmark genes in yellow light’s SGP were related to electron transfer and energy metabolism, such as aldehyde dehydrogenase (NAD+), NADH dehydrogenase and ubiquinol-cytochrome c reductase (Data S6).

## Text S6. Molecular biology mechanism of wavelength-dependent denitrification. We extracted the subcellular location and expression information of PD and phototransduction genes to reconstruct metabolism scheme (Figure S15). Most phototransduction genes up-regulated by blue and yellow light encoded calmodulin (CaM) and G-protein (Figure S6A, Table S2). Light activated G-protein coupled receptor (GPRC) signaled particulate guanylate cyclase (pGC) to transform guanosine triphosphate (GTP) to guanosine monophosphate (cGMP), similarly for cyclic adenosine monophosphate (cAMP) [39]. Both of them were second messengers that transmitted signals through regulatory networks and influenced denitrification [40-42]. Specifically, genes for denitrification were hub genes, involving multiple pathways like two-component system, transporters, and etc. [42] Light signals from receptors of phototransduction were transmitted along the metabolism network to two-component regulatory system, influencing the nitrogen metabolism through nitrogen regulatory kinase NtrB (GlnL) [41]. As shown by the expression levels comparison (Figure S6A), phototransduction was more sensitive to blue light, thus generating more signals to produce diverse metabolites with great potential as active substances [43]. As for denitrification, blue light inhibited the expression of NarK1 but enhanced NarK2 (Dataset S7), two transmembrane proteins for nitrite and nitrate transportation. Therefore, nitrite was stored in the cytoplasm and thus separated from nitrite reductase in the periplasm, explaining the effective nitrite accumulation under blue light (Figure 2A).

## Text S7. Molecular biology mechanism of nitrate-superoxide co-regulation. It was intriguing that microbiota with higher superoxide levels after light exposure enhanced nitrate removal, which was contrary to the common sense on superoxide cytotoxicity [44]. Our co-expression and topological network models succeeded in discovering the nitrate-superoxide correlation mechanism. This was also related to subcellular location. Peroxisome is essential in ROS detoxification and signaling. We extracted all valid DEGs subjected to peroxisome to figure out the nitrate-superoxide correlation mechanism (Dataset S7). SOD was the main enzyme in peroxisome that relieve oxidative stress caused by superoxide. There were several genes encoding SOD. The intracellular SOD (g_41985, Q9RUV2) exhibited the highest expression level. Blue light contributed to higher expression levels of transmembrane SOD (g_29329, P31108), suggesting it scavenged the extracellular superoxide and prevented oxidative stress signaling between cells [28]. Therefore, micro-stimulation of superoxide contributed to inter-cellular signaling to promote substances exchanges and collective cellular metabolism.

## Text S8. Molecular biology mechanism of wavelength-divergent secretion system. Notably, a large proportion of DEGs of secretion system encode pilin secretion and fimbrial assembly proteins, which were up-regulated by yellow light (Dataset S7). This suggested that more pilis were formed in response to illumination for electron transfer and energy metabolism [32]. Blue light significantly up-regulated the expression of Chaperone protein ClpB, one of proteins of type VI secretion system, which is a protein nanomachine that is widespread in Gram-negative bacteria and is used to translocate effector proteins directly into neighboring cells as effective weapons for inter-bacterial competition [45]. The competition was collective responses towards irradiation stress to obtain sufficient resources for survival [46]. Despite unfavorable conditions caused by blue light [24, 42], it was still within the threshold. Microbiota tended to secrete more bioactive substances as inter-cellular signals to maintain homeostasis (Figure S11A), which accelerated the proliferation and evolution of the microbial community, corresponding to the strengthened nitrate removal in self-catalysis experiments (Figure 4F).

## Text S9. Versatility and applications guidance of DMLA cycle. Besides the denitrifying microbiome we showcased in this study, we also conducted extra case studies to better demonstrate the versatility of the DMLA cycle, as well as offer application guidance. The extra case includes the simultaneous CO_2_ fixation-denitrification microbiomes and practical engineering bio-samples from the wastewater treatment plant. Additionally, we also validated the applicability of the proposed methods in other meta-omics like metagenomics. These are stored in https://github.com/YoungeLiao/DMLA. Corresponding analysis can be found in the code base and corresponding HTML file.

Firstly, we demonstrate that our approaches also apply to metagenomics datasets. We conducted metagenomic sequencing on the photo-denitrification microbiome we analyzed in the main texts, and also utilized the geometric deep learning and applications suites in the DMLA cycle to analyze the metagenomic results. The corresponding elucidations are provided alongside the code chunks. For example, in the “APP2: Panel enrichment analysis” section of “DEMO3: Blue light regulated denitrifier based on metagenomics”: “The above gene panel enrichment on cluster 4 unveiled that blue light mainly contribute to activating signals transduction through the two-component system. This is also consistent with the results of metatranscriptomic analysis.”

For CO_2_ fixation-denitrification microbiomes, we use gene definitions to analyze the optogenetic activities under blue light: “It can be clearly observed that cluster 3 was significantly up-regulated, as well as presenting high expression levels under blue light irradiation. Thus, gene panel 3 is our interested functional panel and further analysis will be conducted on it. Enrichment analysis revealed that aldehyde dehydrogenase and inorganic phosphate transporter are two of the most significant genes up-regulated blue light. Under blue light illumination, the transcriptomic activities, especially rRNA, were activated.”

For practical engineering bio-samples from the wastewater treatment plant, enrichment analysis supports the in-lab results well, validating the consistency between in-lab and large scale. We also offered explanations alongside the codebase in Github. For example, in the “APP2: Panel enrichment analysis” section of “DEMO4: Engineering case”: “From the gene panel enrichment results, it can be seen that denitrification microbiome sampled from practical engineering tend to synthesis more EPS, including lipopolysaccharide and exopolysaccharide, which was regulated by two-component system. This is also consistent well with the batch experiments results.”

Supplementary Figures and Tables


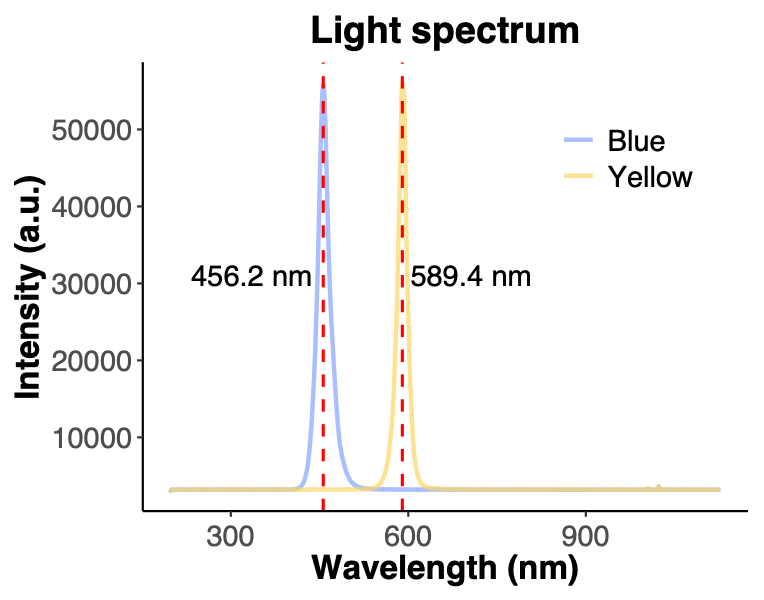


Figure S1. The Light spectrums of LEDs employed to regulate photo-denitrification.


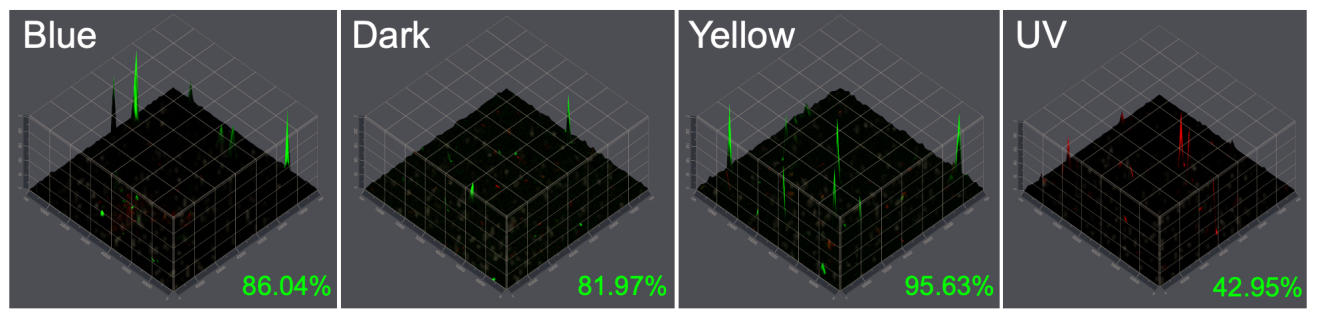


Figure S2. 3D Fluorescence images that depicted the cellular viability and membrane damage. Green: active cells. Red: dead cells. The peak’s height represents fluorescence intensity. The green font percentages represented the ratio of live cells.


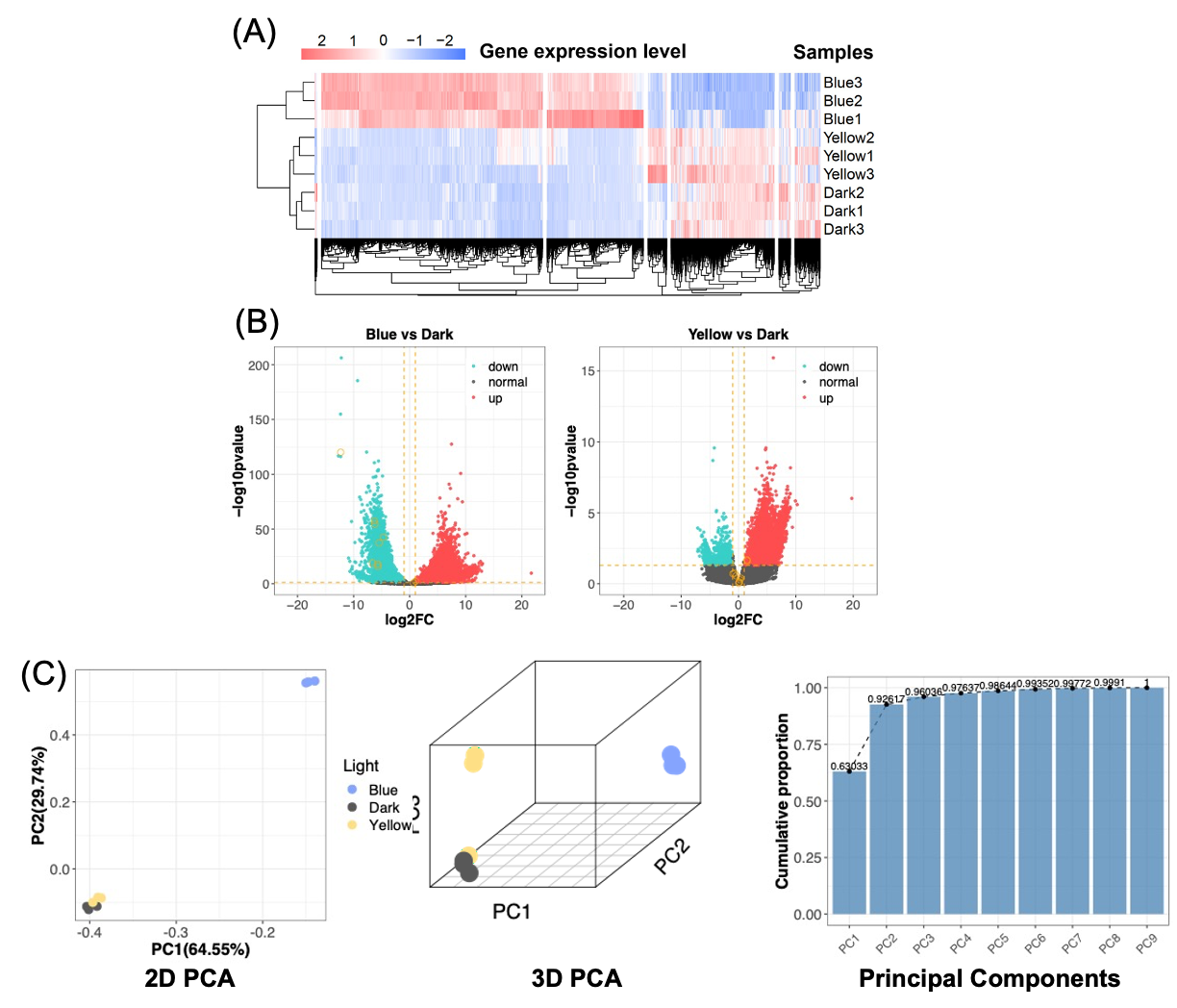


Figure S3. Gene expression profiles and dimension reduction analysis. (A) Gene expression patterns of valid DEGs. The expression levels were log normalized and scaled based on FPKM. (B) Volcano plots of the DESeq results of blue and yellow lights. The dark condition was utilized as control. The orange cycle highlighted the top 10 highly-expressed genes. (C) Dimension reduction through principal components analysis (PCA).


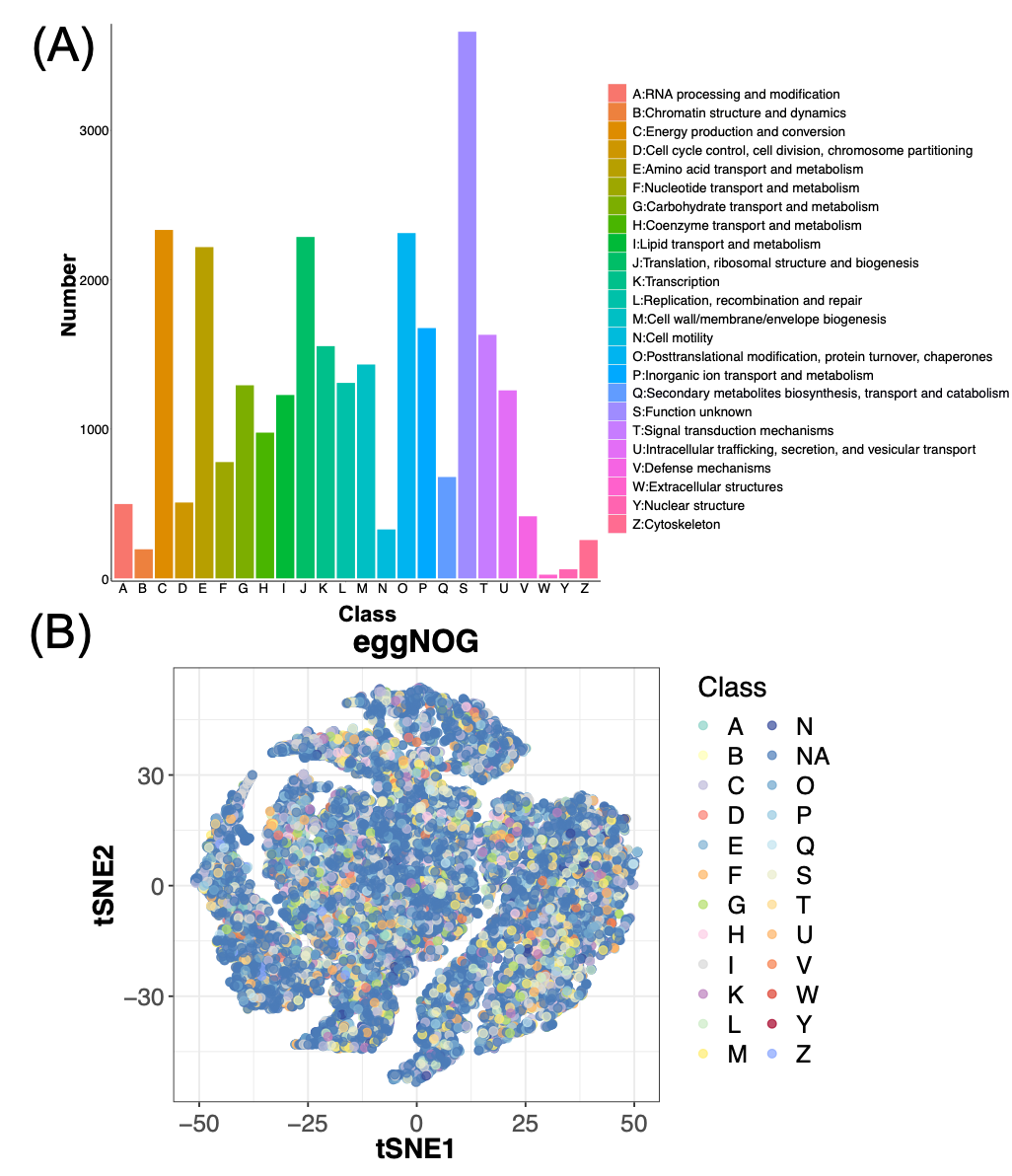


Figure S4. EggNOG class distribution on all differentially expressed genes (DEGs). (A) Genes counts that assigned to different eggNOG class. Class abbreviation and more information see Supplementary Table 2. (B) Gene expression distribution and corresponding eggnog labels of valid DEGs after dimension reduction through tSNE. NA denotes genes lack of eggNOG annotation.


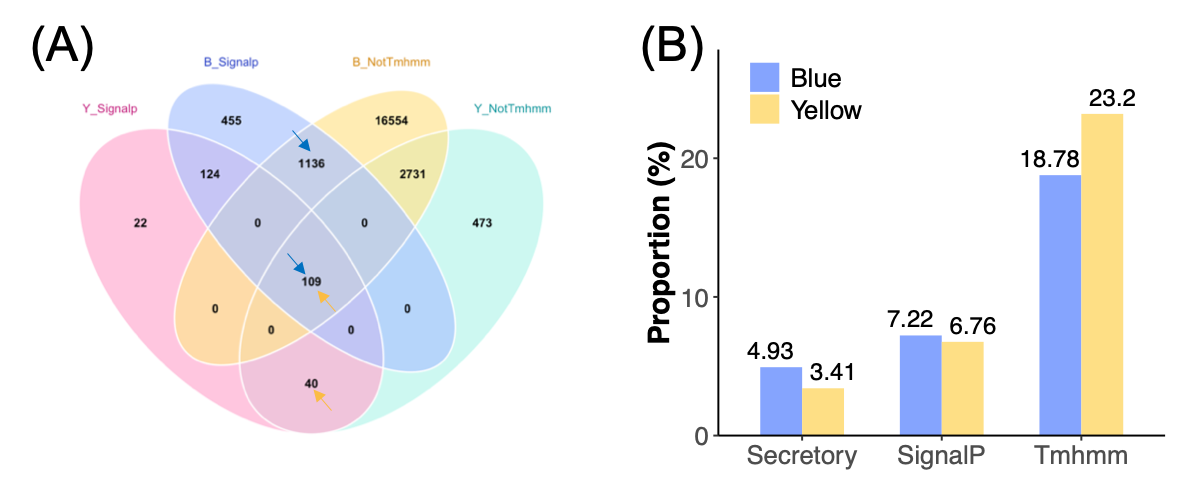


Figure S5. Subcellular location profiles of blue light’s and yellow light’s valid DEGs. (A) Blue and yellow errors denoted secretory protein responsive to blue and yellow light. B_Signalp and Y_Signalp represent DEGs that encoded signal peptides of blue and yellow light. B_NotTmhmm and Y_NotTmhmm represent DEGs that encode protein without transmembrane domains of blue and yellow light. The overlaps of Signalp and NotTmhmm were secretory proteins. (B) Proportion of secreted protein, signal peptide and transmembrane protein in DEGs of blue and yellow light.


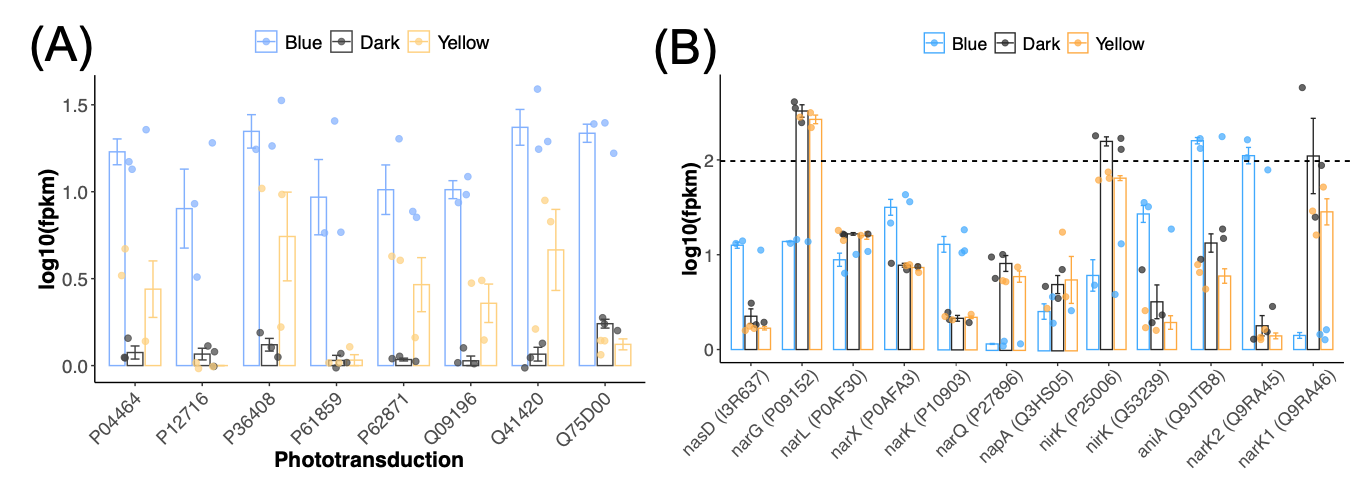


Figure S6. Expression patterns of major DEGs that related to phototransduction and nitrate conversion. (A) Expression levels of DEGs that annotated to phototransduction. Genes were denoted by Swissprot ID. Functional information can be found in Table S2. (B) Expression levels of nitrate- and nitrite-related genes, namely PD genes. Genes with expression above the dash line were regarded as highly-expressed genes. See Table S3 for details. *expre*: expression level (FPKM).


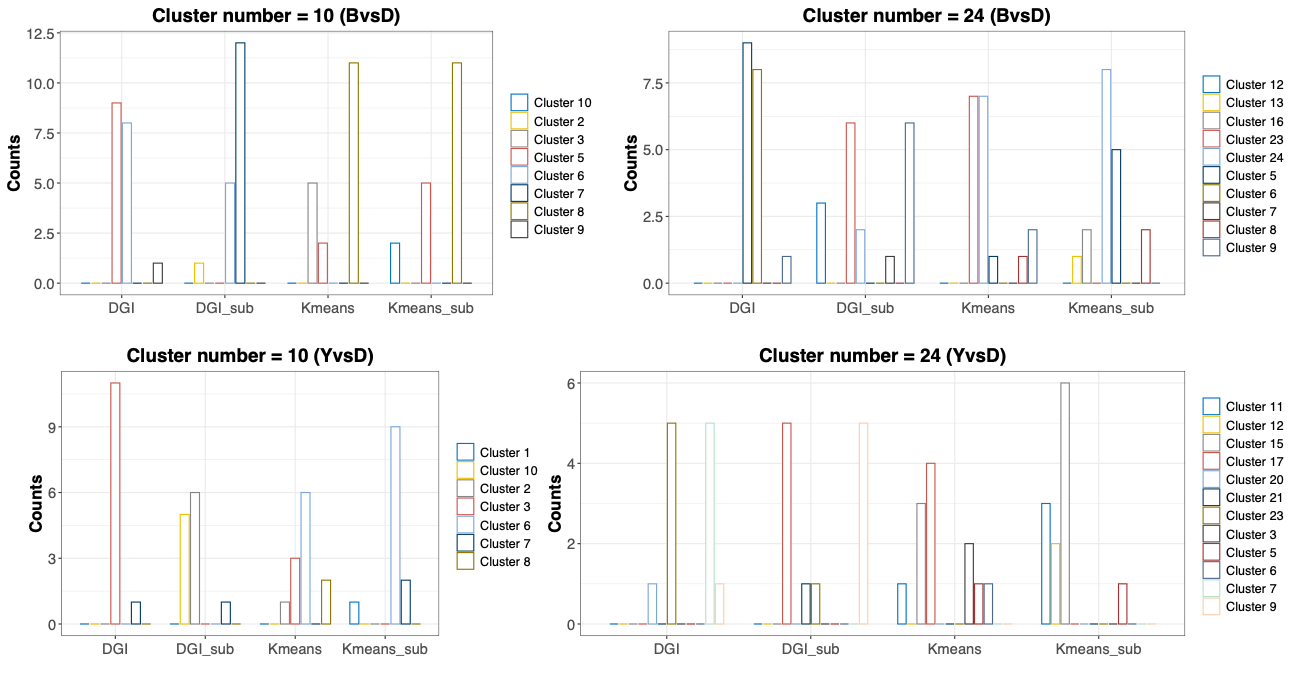


Figure S7. Cluster assignment of phototransduction genes under different cluster number. “Blue vs Dark”: DEGs of blue light group. “Yellow vs Dark”: DEGs of yellow light group.

Figure S8. Cluster distribution of functional pathways. Nitrogen: nitrogen metabolism. Xebio: metabolism of xenobiotics by cytochrome P450.

Figure S9. Pathway enrichment analysis on significant photo-denitrification pathways of blue (A) and yellow light (B). Fold changes were calculated with the dark group as control. Expression was the mean gene expression under blue or yellow light.


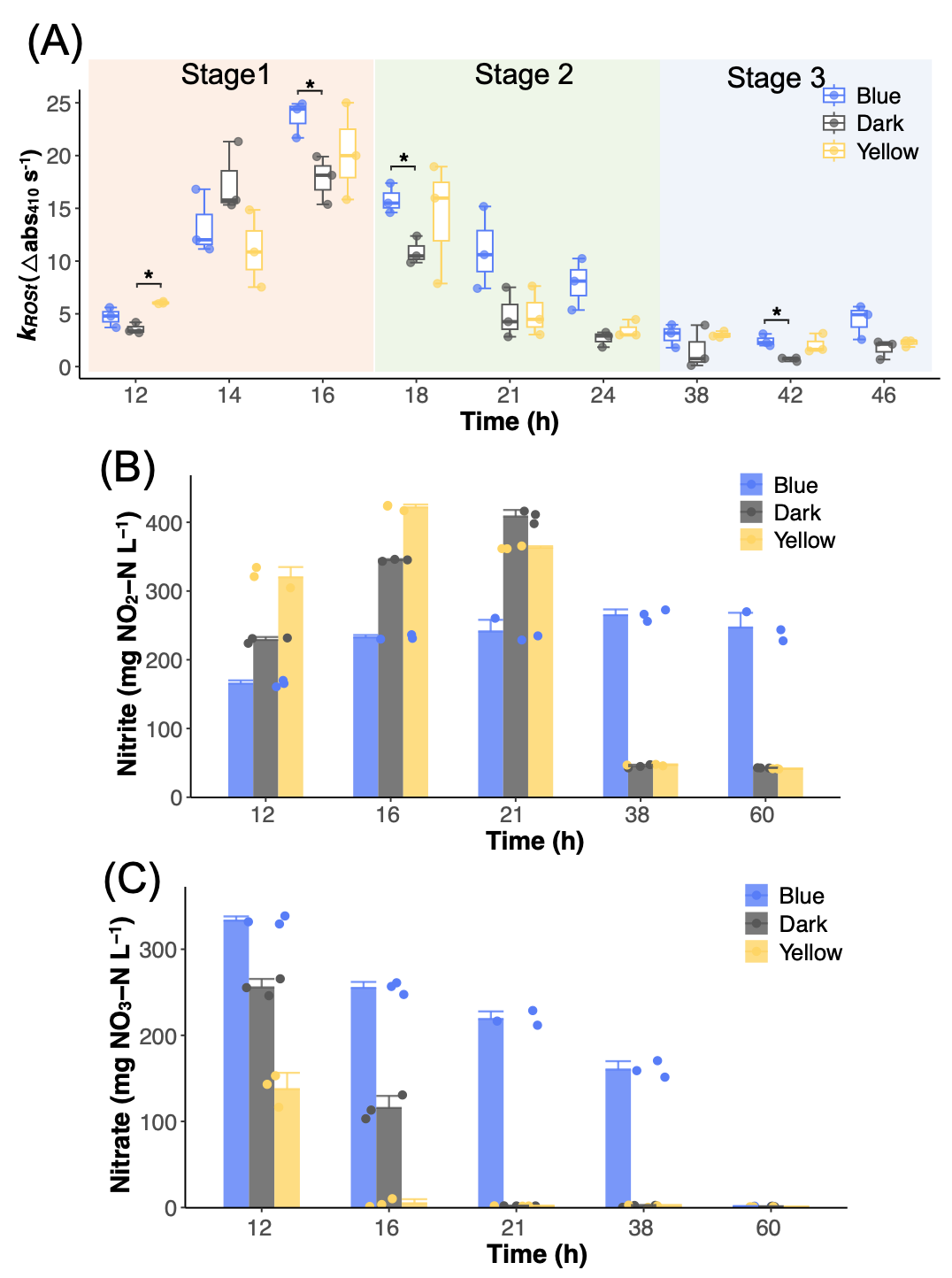


Figure S10. Wet-lab validations on co-expression between total reactive oxygen species (ROS) levels and photo-denitrification. (A) Total ROS variation in the time course. Photo-denitrification was divided into three stages based on main nitrogen metabolism. Stage 1: nitrate reduction. Stage 2: nitrite reduction. Stage 3: Inorganic nitrogen depletion. Nitrite (B) and nitrate (C) concentrations variation during photo-denitrification. It can be observed that total ROS shared similar trends with nitrite concentrations.


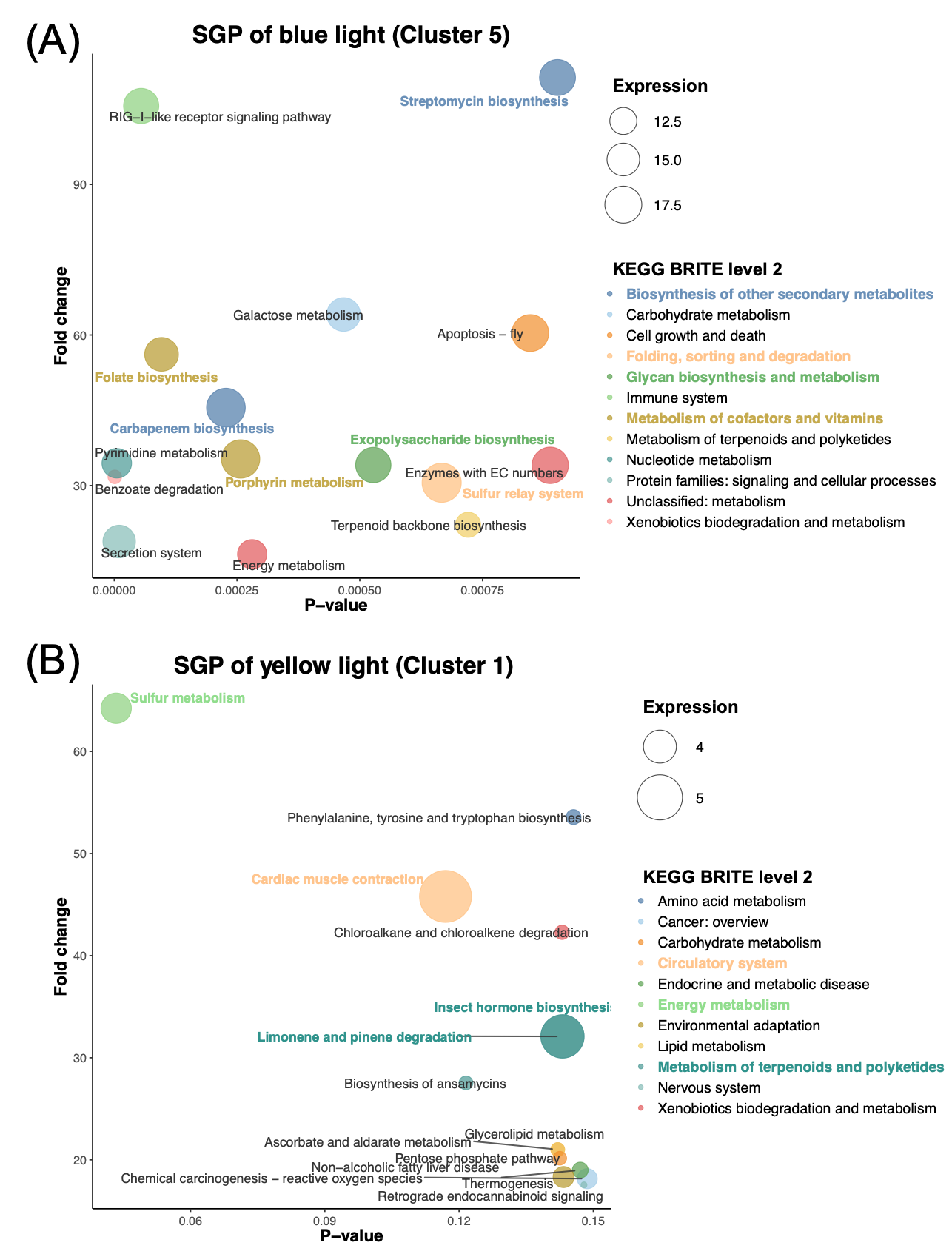


Figure S11. Pathways enrichment analysis of signaling gene panels (SGPs) of blue (A) and yellow (B) light. The most highly expressed pathways (Table S4) and their KEGG Brite was highlighted by corresponding color and bold font. Bubble size denoted mean expression levels (FPKM) under blue or yellow light, respectively. Fold changes were calculated with the dark group as control.


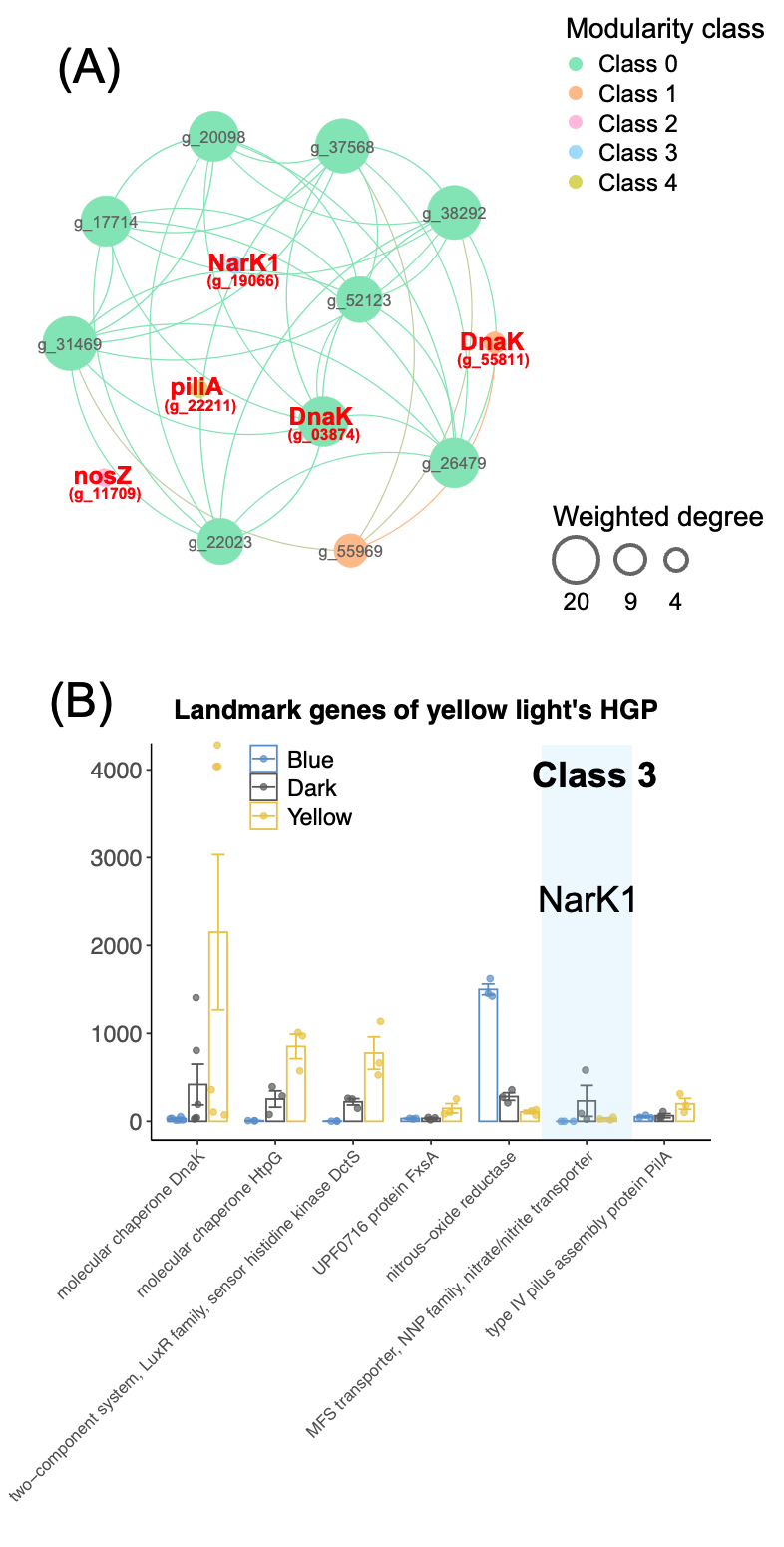


Figure S12. The topological network model and corresponding landmark genes of yellow light’s HGP. (A) The gene topological network. Details on the topological information of gene nodes and landmark genes were summarized in Dataset S2 and S6. The bold red font highlighted the landmark genes with the highest expression. The bold black font highlighted the principal denitrification genes. (B) Expression levels of landmark genes. Background highlighted the modularity class PD and phototransduction genes subjected to.


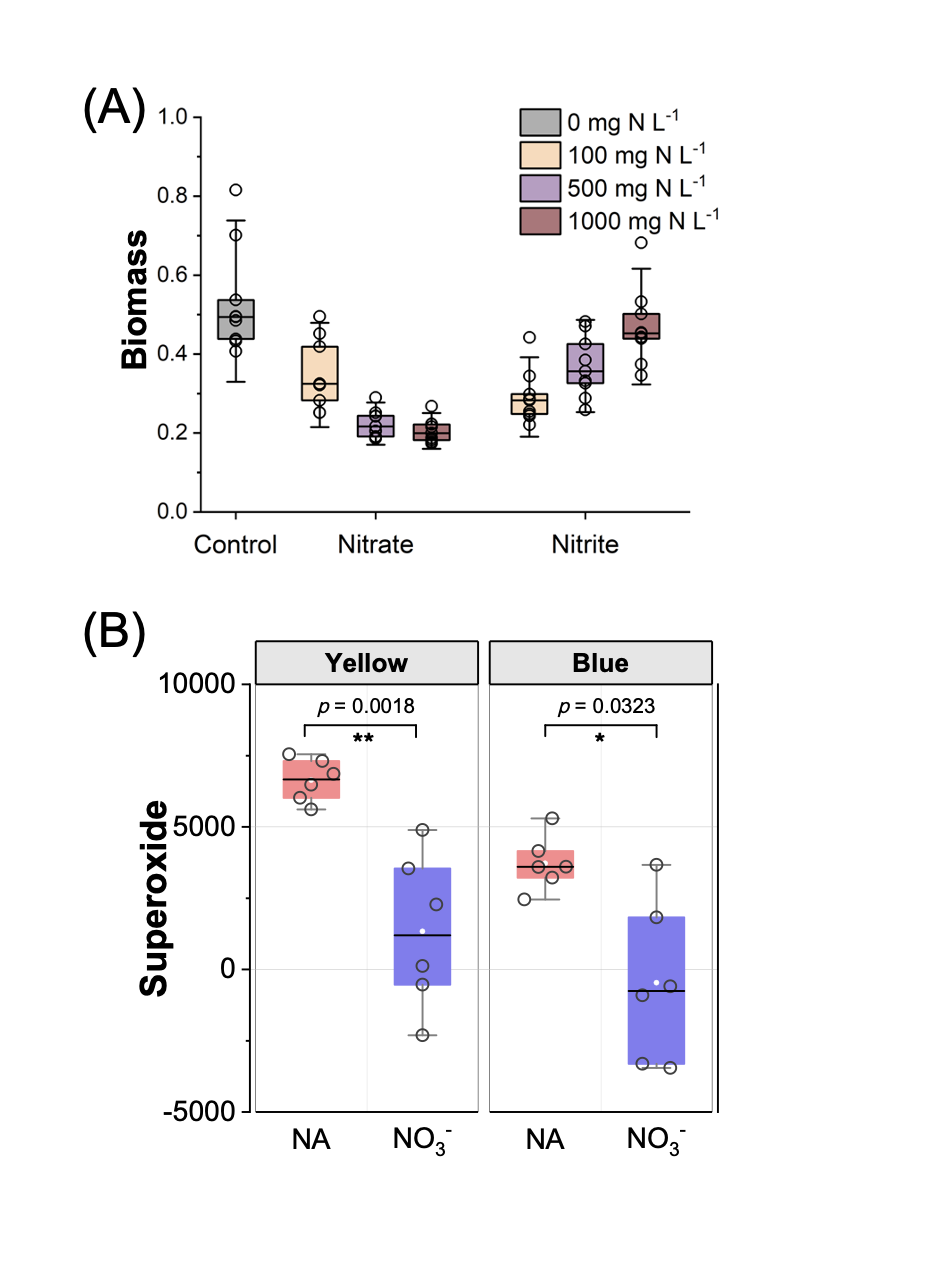


Figure S13. Co-expression of nitrate metabolism and superoxide production. (A) Biomass synthesis under different nitrate and nitrite concentrations. (B) Influence of nitrate absence on superoxide level under yellow and blue light.


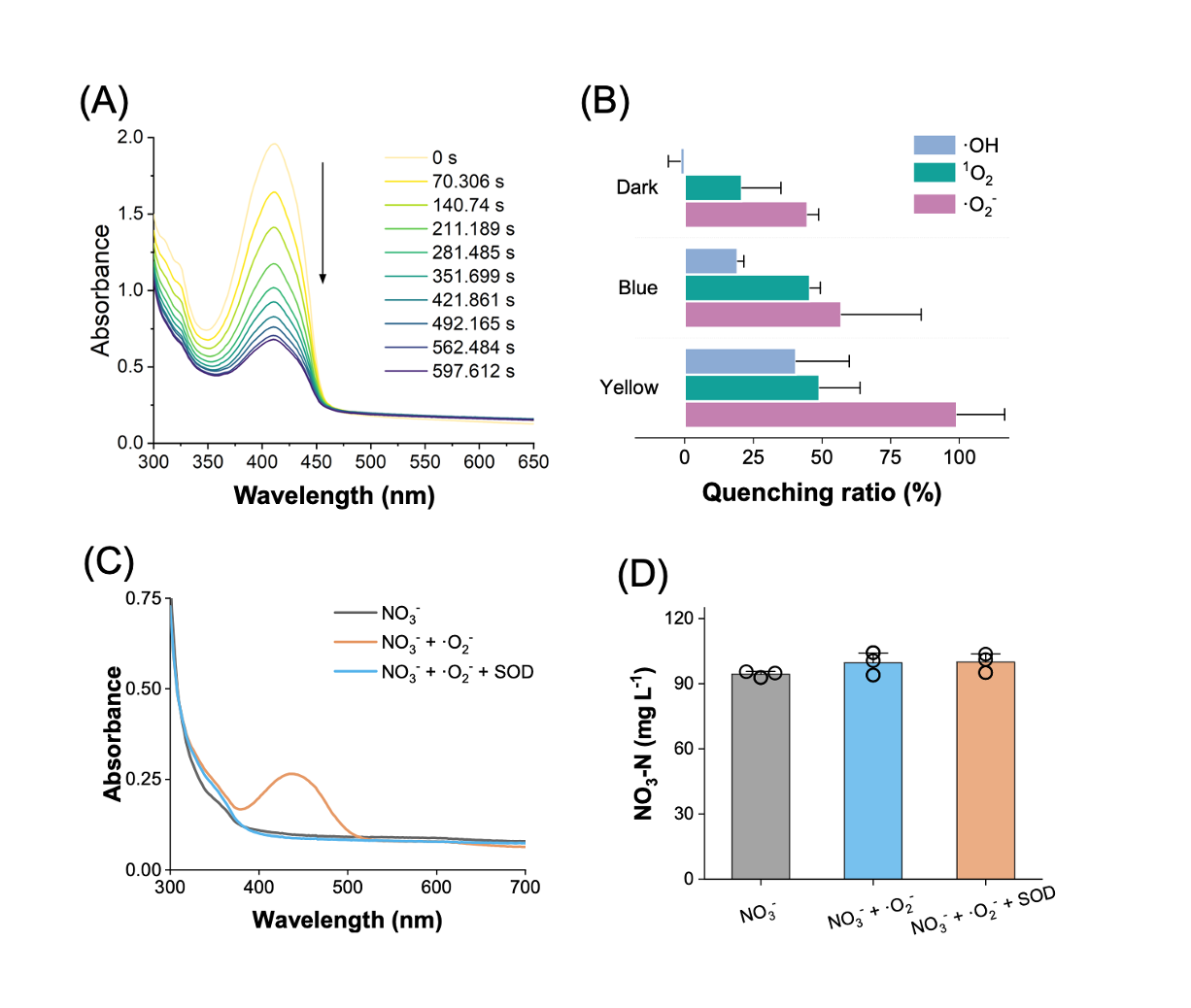


Figure S14. ROS assay and addition of superoxide. (A) Dynamic total ROS production under dark condition. (B) Quenching experiments under different illumination conditions. Quenching ratio was calculated based on nitrate removal efficiency. (C) Detection of superoxide added by biological methods. (D) Non-biological reactions between nitrate, superoxide and SOD.

Figure S15. Mechanistic scheme of light-regulated denitrification. It was reconstructed based on the subcellular location of critical enzymes involved in phototransduction and nitrogen metabolism. CaM: Calmodulin.  GPCR: G-protein coupled receptor. CNG ion channel: cyclic nucleotide-gated ion channel. For details on genes and protein abbreviations see Table S2, S3.

Legends for Datasets

## Dataset S1. Topological properties of blue light's hub gene panels (HGP).

## Dataset S2. Topological properties of yellow light's hub gene panels (HGP).

## Dataset S3. Topological properties of blue light's signaling gene panel (SGP).

## Dataset S4. Topological properties of yellow light's signaling gene panel (SGP).

## Dataset S5. Landmark genes of blue light.

## Dataset S6. Landmark genes of yellow light.

## Dataset S7. Subcellular information and functional annotations of critical light-responsive genes.

Supplementary References

1. Kopylova, Evguenia, Laurent Noé, Hélène Touzet. 2012. “SortMeRNA: fast and accurate filtering of ribosomal RNAs in metatranscriptomic data.” *Bioinformatics* 28: 3211-3217. <https://doi.org/10.1093/bioinformatics/bts611>

2. Grabherr, Manfred G., Brian J. Haas, Moran Yassour, Joshua Z. Levin, Dawn A. Thompson, Ido Amit, Xian Adiconis, et al. 2011. “Trinity: reconstructing a full-length transcriptome without a genome from RNA-Seq data.” *Nature Biotechnology* 29: 644-652. <https://doi.org/10.1038/nbt.1883>

3. Ismail, Wazim Mohammed, Yuzhen Ye, Haixu Tang. 2014. “Gene finding in metatranscriptomic sequences.” *BMC Bioinformatics* 15: S8. <https://doi.org/10.1186/1471-2105-15-S9-S8>

4. Fu, Limin, Beifang Niu, Zhengwei Zhu, Sitao Wu, Weizhong Li. 2012. “CD-HIT: accelerated for clustering the next-generation sequencing data.” *Bioinformatics* 28: 3150-3152. <https://doi.org/10.1093/bioinformatics/bts565>

5. Li, Bo, Colin N. Dewey. 2011. “RSEM: accurate transcript quantification from RNA-Seq data with or without a reference genome.” *BMC Bioinformatics* 12: 323. <https://doi.org/10.1186/1471-2105-12-323>

6. Altschul, Stephen F., Thomas L. Madden, Alejandro A. Schäffer, Jinghui Zhang, Zheng Zhang, Webb Miller, David J. Lipman. 1997. “Gapped BLAST and PSI-BLAST: a new generation of protein database search programs.” *Nucleic acids research* 25: 3389-3402. <https://doi.org/10.1093/nar/25.17.3389>

7. Lalwani, Makoto, Samantha Ip, César Carrasco-López, Catherine Day, Evan Zhao, Hinako Kawabe, José Avalos. 2021. “Optogenetic control of the lac operon for bacterial chemical and protein production.” *Nature Chemical Biology* 17: 1-9. <https://doi.org/10.1038/s41589-020-0639-1>

8. Cao, Zhi-Jie, Ge Gao. 2022. “Multi-omics single-cell data integration and regulatory inference with graph-linked embedding.” *Nature Biotechnology* 40: 1458-1466. <https://doi.org/10.1038/s41587-022-01284-4>

9. Ribeiro, Diogo M., Simone Rubinacci, Anna Ramisch, Robin J. Hofmeister, Emmanouil T. Dermitzakis, Olivier Delaneau. 2021. “The molecular basis, genetic control and pleiotropic effects of local gene co-expression.” *Nature Communications* 12: 4842. <https://doi.org/10.1038/s41467-021-25129-x>

10. Huerta-Cepas, Jaime, Damian Szklarczyk, Davide Heller, Ana Hernández-Plaza, Sofia K Forslund, Helen Cook, Daniel R Mende, et al. 2018. “eggNOG 5.0: a hierarchical, functionally and phylogenetically annotated orthology resource based on 5090 organisms and 2502 viruses.” *Nucleic acids research* 47: D309-D314. <https://doi.org/10.1093/nar/gky1085>

11. Subramanian, Aravind, Rajiv Narayan, Steven M. Corsello, David D. Peck, Ted E. Natoli, Xiaodong Lu, Joshua Gould, et al. 2017. “A Next Generation Connectivity Map: L1000 Platform and the First 1,000,000 Profiles.” *Cell* 171: 1437-1452.e17. <https://doi.org/10.1016/j.cell.2017.10.049>

12. Gelman, Sam, Sarah A. Fahlberg, Pete Heinzelman, Philip A. Romero, Anthony Gitter. 2021. “Neural networks to learn protein sequence–function relationships from deep mutational scanning data.” *Proceedings of the National Academy of Sciences* 118: e2104878118. <https://doi.org/10.1073/pnas.2104878118>

13. Koppel, Nitzan, Vayu Rekdal, Emily Balskus. 2017. “Chemical transformation of xenobiotics by the human gut microbiota.” *Science* 356: 1246-1257. <https://doi.org/10.1126/science.aag2770>

14. Kuypers, Marcel MM, Hannah K Marchant, Boran Kartal. 2018. “The microbial nitrogen-cycling network.” *Nature Reviews Microbiology* 16: 263-276. <https://doi.org/10.1038/nrmicro.2018.9>

15. Vogel, Lauren. 2019. “Health workers demand health coverage for migrants.” *CMAJ : Canadian Medical Association Journal* 191: E270. <https://doi.org/10.1503/cmaj.109-5721>

16. Salas-Pérez, Francisca, Omar Ramos-Lopez, María L. Mansego, Fermín I. Milagro, José L. Santos, José I. Riezu-Boj, J. Alfredo Martínez. 2019. “DNA methylation in genes of longevity-regulating pathways: association with obesity and metabolic complications.” *Aging (Albany NY)* 11: 1874-1899. <https://doi.org/10.18632/aging.101882>

17. Egan, Alexander J. F., Jeff Errington, Waldemar Vollmer. 2020. “Regulation of peptidoglycan synthesis and remodelling.” *Nature Reviews Microbiology* 18: 446-460. <https://doi.org/10.1038/s41579-020-0366-3>

18. Tu-Sekine, Becky, Sangwon F. Kim. 2022. “The Inositol Phosphate System-A Coordinator of Metabolic Adaptability.” *International Journal of Molecular Sciences* 23: 6747. <https://doi.org/10.3390/ijms23126747>

19. Majumdar, S. K., H. J. Kutzner. 1962. “Myo-inositol in the Biosynthesis of Streptomycin by Streptomyces griseus.” *Science* 135: 734-734. <https://doi.org/10.1126/science.135.3505.734>

20. Singhal, Sonia. 2020. “Digest: Structuring interactions in Streptomyces*.” *Evolution* 74: 207-209. <https://doi.org/10.1111/evo.13874>

21. Pegtel, D. Michiel, Stephen J. Gould. 2019. “Exosomes.” *Annual Review of Biochemistry* 88: 487-514. <https://doi.org/10.1146/annurev-biochem-013118-111902>

22. Zhang, Yuan, Yunfeng Liu, Haiying Liu, Wai Ho Tang. 2019. “Exosomes: biogenesis, biologic function and clinical potential.” *Cell & Bioscience* 9: 19. <https://doi.org/10.1186/s13578-019-0282-2>

23. Chai, Yunrong, Pascale B. Beauregard, Hera Vlamakis, Richard Losick, Roberto Kolter. 2012. “Galactose metabolism plays a crucial role in biofilm formation by Bacillus subtilis.” *mBio* 3: e00184-00112. <https://doi.org/10.1128/mBio.00184-12>

24. Bumah, Violet Vakunseh, Brianna Nicole Morrow, Paulina Michelle Cortez, Chynna Rose Bowman, Paulina Rojas, Daniela Santos Masson-Meyers, James Suprapto, William G. Tong, Chukuka Samuel Enwemeka. 2020. “The importance of porphyrins in blue light suppression of Streptococcus agalactiae.” *Journal of Photochemistry and Photobiology B: Biology* 212: 111996. <https://doi.org/10.1016/j.jphotobiol.2020.111996>

25. Schmid, Jochen, Volker Sieber, Bernd Rehm. 2015. “Bacterial exopolysaccharides: biosynthesis pathways and engineering strategies.” *Frontiers in Microbiology* 6: 496. <https://doi.org/10.3389/fmicb.2015.00496>

26. Dahl, Christiane, Rüdiger Hell, Thomas Leustek, David Knaff. 2008. Introduction to Sulfur Metabolism in Phototrophic Organisms. *Sulfur Metabolism in Phototrophic Organisms* Springer Netherlands, 1-14. <https://doi.org/10.1007/978-1-4020-6863-8_1>

27. Zha, Lingyan, Wenke Liu, Qichang Yang, Yubin Zhang, Chengbo Zhou, Mingjie Shao. 2020. “Regulation of Ascorbate Accumulation and Metabolism in Lettuce by the Red:Blue Ratio of Continuous Light Using LEDs.” *Frontiers in Plant Science* 11: <https://doi.org/10.3389/fpls.2020.00704>

28. Apel, Klaus, Heribert Hirt. 2004. “Reactive Oxygen Species: Metabolism, Oxidative Stress, and Signal Transduction.” *Annual review of plant biology* 55: 373-399. <https://doi.org/10.1146/annurev.arplant.55.031903.141701>

29. Helvig, C., J. F. Koener, G. C. Unnithan, R. Feyereisen. 2004. “CYP15A1, the cytochrome P450 that catalyzes epoxidation of methyl farnesoate to juvenile hormone III in cockroach corpora allata.” *Proceedings of the National Academy of Sciences* 101: 4024-4029. <https://doi.org/10.1073/pnas.0306980101>

30. Jurenka, Russell. 2004. “Insect pheromone biosynthesis.” *Topics in Current Chemistry* 239: 97-132. <https://doi.org/10.1007/b95450>

31. Ni, Jianguo, Huayun Yang, Liqing Chen, Jiadong Xu, Liangwei Zheng, Guojian Xie, Chenjia Shen, Weidong Li, Qi Liu. 2022. “Metagenomic analysis of microbial community structure and function in a improved biofilter with odorous gases.” *Scientific Reports* 12: 1731. <https://doi.org/10.1038/s41598-022-05858-9>

32. Lovley, Derek R. 2017. “Syntrophy Goes Electric: Direct Interspecies Electron Transfer.” *Annual Review of Microbiology* 71: 643-664. <https://doi.org/10.1146/annurev-micro-030117-020420>

33. Chen, Can, Keqi Chen, Tao Su, Bing Zhang, Guizhi Li, Junfeng Pan, Meiru Si. 2019. “Myo-inositol-1-phosphate synthase (Ino-1) functions as a protection mechanism in Corynebacterium glutamicum under oxidative stress.” *MicrobiologyOpen* 8: e00721. <https://doi.org/10.1002/mbo3.721>

34. Schmitz, Rob A., Andreas Dietl, Melanie Müller, Tom Berben, Huub J. M. Op den Camp, Thomas R. M. Barends. 2020. “Structure of the 4-hydroxy-tetrahydrodipicolinate synthase from the thermoacidophilic methanotroph Methylacidiphilum fumariolicum SolV and the phylogeny of the aminotransferase pathway.” *Acta Crystallographica. Section F, Structural Biology Communications* 76: 199-208. <https://doi.org/10.1107/S2053230X20005294>

35. Zeng, Li-Rong, Jian-Ping Xie. 2011. “Molecular basis underlying LuxR family transcription factors and function diversity and implications for novel antibiotic drug targets.” *Journal of Cellular Biochemistry* 112: 3079-3084. <https://doi.org/10.1002/jcb.23262>

36. Zhong, Xiaojun, Ranran Lu, Fuwen Liu, Jinjie Ye, Junyang Zhao, Fei Wang, Menghua Yang. 2021. “Identification of LuxR Family Regulators That Integrate Into Quorum Sensing Circuit in Vibrio parahaemolyticus.” *Frontiers in Microbiology* 12, <https://www.frontiersin.org/articles/10.3389/fmicb.2021.691842>

37. Balasaheb Nimse, Satish, Dilipkumar Pal. 2015. “Free radicals, natural antioxidants, and their reaction mechanisms.” *RSC Advances* 5: 27986-28006. <https://doi.org/10.1039/C4RA13315C>

38. Chen, Y. T., R. L. Zheng, Z. J. Jia, Y. Ju. 1990. “Flavonoids as superoxide scavengers and antioxidants.” *Free Radical Biology & Medicine* 9: 19-21. <https://doi.org/10.1016/0891-5849(90)90045-k>

39. Johnson, Jacque-Lynne F., Michel R. Leroux. 2010. “cAMP and cGMP signaling: sensory systems with prokaryotic roots adopted by eukaryotic cilia.” *Trends in Cell Biology* 20: 435-444. <https://doi.org/10.1016/j.tcb.2010.05.005>

40. Emiliani, Valentina, Emilia Entcheva, Rainer Hedrich, Peter Hegemann, Kai R Konrad, Christian Lüscher, Mathias Mahn, Zhuo-Hua Pan, Ruth R Sims, Johannes Vierock. 2022. “Optogenetics for light control of biological systems.” *Nature Reviews Methods Primers* 2: 55. <https://doi.org/10.1038/s43586-022-00136-4>

41. Sanders, D. A., B. L. Gillece-Castro, A. L. Burlingame, D. E. Koshland. 1992. “Phosphorylation site of NtrC, a protein phosphatase whose covalent intermediate activates transcription.” *Journal of Bacteriology* 174: 5117-5122. <https://doi.org/10.1128/jb.174.15.5117-5122.1992>

42. Swartz, Trevor E., Tong-Seung Tseng, Marcus A. Frederickson, Gastón Paris, Diego J. Comerci, Gireesh Rajashekara, Jung-Gun Kim, et al. 2007. “Blue-Light-Activated Histidine Kinases: Two-Component Sensors in Bacteria.” *Science* 317: 1090-1093. <https://doi.org/10.1126/science.1144306>

43. Liao, Yang, Jiyong Bian, Shiyu Miao, Siqi Xu, Rui Li, Ruiping Liu, Huijuan Liu, Jiuhui Qu. 2023. “Regulation of denitrification performance and microbial topology by lights: Insight into wavelength effects towards microbiota.” *Water Research* 232: 119434. <https://doi.org/10.1016/j.watres.2022.119434>

44. Georgiou, Christos D., Henry J. Sun, Christopher P. McKay, Konstantinos Grintzalis, Ioannis Papapostolou, Dimitrios Zisimopoulos, Konstantinos Panagiotidis, et al. 2015. “Evidence for photochemical production of reactive oxygen species in desert soils.” *Nature Communications* 6: 7100. <https://doi.org/10.1038/ncomms8100>

45. Hernandez, Ruth E., Ramses Gallegos-Monterrosa, Sarah J. Coulthurst. 2020. “Type VI secretion system effector proteins: Effective weapons for bacterial competitiveness.” *Cellular Microbiology* 22: e13241. <https://doi.org/10.1111/cmi.13241>

46. Lee, Sukyeong, Mathew E. Sowa, Yo-hei Watanabe, Paul B. Sigler, Wah Chiu, Masasuke Yoshida, Francis T. F. Tsai. 2003. “The structure of ClpB: a molecular chaperone that rescues proteins from an aggregated state.” *Cell* 115: 229-240. <https://doi.org/10.1016/s0092-8674(03)00807-9>
